# Phyllostomid Bat Occurrence in Successional Stages of Neotropical Dry Forests

**DOI:** 10.1371/journal.pone.0084572

**Published:** 2014-01-03

**Authors:** Luis Daniel Avila-Cabadilla, Kathryn Elizabeth Stoner, Jafet M. Nassar, Mario M. Espírito-Santo, Mariana Yolotl Alvarez-Añorve, Carla I. Aranguren, Mickael Henry, José A. González-Carcacía, Luiz A. Dolabela Falcão, Gerardo Arturo Sanchez-Azofeifa

**Affiliations:** 1 Escuela Nacional de Estudios Superiores, Unidad Morelia, Universidad Nacional Autónoma de México, Morelia, México; 2 Centro de Investigaciones en Ecosistemas, Universidad Nacional Autónoma de México, Morelia, México; 3 Department of Fish, Wildlife and Conservation Ecology, New Mexico State University, Las Cruces, New Mexico, United States of America; 4 Centro de Ecología, Instituto Venezolano de Investigaciones Científicas Altos de Pipe, Caracas, Venezuela; 5 Departamento de Biologia Geral, Universidade Estadual de Montes Claros, Montes Claros, Minas Gerais, Brazil; 6 INRA (Institut National de la Recherche Agronomique), UR 406 Abeilles & Environnement, Site Agroparc, Avignon, France; 7 Earth and Atmospheric Sciences Department, University of Alberta, Edmonton, Canada; Università degli Studi di Napoli Federico II, Italy

## Abstract

Tropical dry forests (TDFs) are highly endangered tropical ecosystems being replaced by a complex mosaic of patches of different successional stages, agricultural fields and pasturelands. In this context, it is urgent to understand how taxa playing critical ecosystem roles respond to habitat modification. Because Phyllostomid bats provide important ecosystem services (e.g. facilitate gene flow among plant populations and promote forest regeneration), in this study we aimed to identify potential patterns on their response to TDF transformation in sites representing four different successional stages (initial, early, intermediate and late) in three Neotropical regions: México, Venezuela and Brazil. We evaluated bat occurrence at the species, ensemble (abundance) and assemblage level (species richness and composition, guild composition). We also evaluated how bat occurrence was modulated by the marked seasonality of TDFs. In general, we found high seasonal and regional specificities in phyllostomid occurrence, driven by specificities at species and guild levels. For example, highest frugivore abundance occurred in the early stage of the moistest TDF, while highest nectarivore abundance occurred in the same stage of the driest TDF. The high regional specificity of phyllostomid responses could arise from: (1) the distinctive environmental conditions of each region, (2) the specific behavior and ecological requirements of the regional bat species, (3) the composition, structure and phenological patterns of plant assemblages in the different stages, and (4) the regional landscape composition and configuration. We conclude that, in tropical seasonal environments, it is imperative to perform long-term studies considering seasonal variations in environmental conditions and plant phenology, as well as the role of landscape attributes. This approach will allow us to identify potential patterns in bat responses to habitat modification, which constitute an invaluable tool for not only bat biodiversity conservation but also for the conservation of the key ecological processes they provide.

## Introduction

In the Neotropics, the natural landscape has been increasingly modified by human activities such as cattle raising and agriculture [1]–[3]. This has provoked the replacement of natural vegetation by a complex mosaic of patches representing different degrees of regeneration, agricultural fields and pasturelands [4], [5]. In fact, in several countries that present more than 2% of annual deforestation [2], transformed landscape will likely be the predominant habitat available for wildlife in the near future [5]. Consequently, it is urgent to determine which factors shape the distribution and performance of biota in Neotropical landscapes transformed by human activities, paying special attention to taxa participating in critical ecosystem functioning [6], [7].

Bats are considered one of the key-stone groups in the Neotropics due to the ecological services they provide: seed dispersal, pollination, control of invertebrate and small vertebrate populations and recycling and translocation of nutrients and energy [8]–[10]. Bats favor the maintenance of plant diversity as they promote outcrossing, facilitate gene flow among distant plant populations and make possible dispersion of plant species across landscapes, via pollen and seed translocation [8], [11], [12]. In depauperate tropical areas it has been suggested that bats can promote forest regeneration as nearly half of the most abundant pioneer plants are dispersed by them (e.g. genera *Solanum*, *Cecropia*, *Piper*, *Vismia*) [13]. In addition, insectivorous bats can significantly reduce forest herbivory levels as well [10].

One of the most endangered ecosystems within the Neotropics is the tropical dry forest (TDF), a preferred zone for agriculture and human settlements [3], [4], which is distributed along most of the latitudinal gradient encompassing the Neotropics (approximate extension 519,597 km^2^) [14]. In spite of being one of the most endangered ecosystems (66% reduction in its original area), TDF is one of the least protected habitats and historically has received little scientific attention in comparison to other Neotropical systems [4], [14].

Tropical dry forests are characterized by a marked seasonality in the precipitation regime and a severe dry season (DS), which can last from three to eight months, depending on the latitudinal position (TDFs farthest from the equator present the longest DS) [4], [15]. Due to the seasonality in the precipitation regime at least 50% of trees found in TDF are deciduous and drop their leaves during the DS [4], [16]. As plant phenological patterns and primary productivity are strongly seasonal in TDFs, bats occurring in these systems have to deal with significant seasonal changes in terms of vegetation structure and type and amount of food. Kalacska et al. [17], for example, report significant differences between seasons in canopy openness, plant area index (PAI) and leaf area index (LAI) of three TDFs. These changes in vegetation structure could significantly impact the activity of several bat species found in the Neotropics, especially those belonging to the family Phyllostomidae, which strongly depend on highly cluttered spaces for foraging and are sensitive to changes in vegetation structure [6], [18]. Nectarivorous and frugivorous bats face significant variations in food availability, due to the pronounced seasonality in the flowering and fruiting of trees and shrubs [19], [20]. Both aerial and gleaning insectivores also can experience significant changes in food availability between seasons as precipitation, primary productivity and insect abundance and diversity are positively related in the tropics [21], [22].

In general, tropical bats deal with seasonal fluctuations of key resources (i.e. food, roost) and climate conditions (i.e. humidity, temperature) through behavioral and physiological adaptations. Examples of this are changes in diet breadth, type of food, food intake rate, patterns of habitat use, defense of feeding areas and migratory behavior [23]–[27]. Indeed, tropical bats have the capacity to enter in torpor as a response to energetic restrictions and as a mechanism for avoiding dehydration in arid zones [28], [29].

The response of bats to seasonality provokes marked seasonal changes in their assemblages. Stoner [20], [30], [31] and Avila-Cabadilla et al. [32], for example, found significant changes in abundance of frugivorous and nectarivorous bats between seasons in TDFs. In spite of this seasonal variation in bat tropical assemblages, most of the studies addressing bat response to habitat alteration have combined data across seasons or analyzed data from a single season [33]. These approaches may hide major response patterns at the assemblage level. Hereafter, we will use the terms assemblage and ensemble *sensu* Fauth et al. [34], who recognized assemblage as a phylogenetically bounded group of species inhabiting a given local habitat (e.g. phyllostomid assemblage) and ensemble as a set of species within an assemblage that use a similar set of resources (e.g. phyllostomid frugivores).

In spite of the variable methodologies employed for studying bat response to habitat modification, some patterns emerge in the literature. For example, rare species with specialized diet and habitat requirements, such as gleaning insectivores and carnivores from the subfamily Phyllostominae, are more tightly associated with mature forest [35]–[38] than secondary forest. In the case of frugivores in tropical humid forests, bat abundance is higher in anthropogenic habitats than in mature forests, which is linked to an increase in the abundance of pioneer chiropterochoric plants [35], [36]. In general, species less affected by habitat modification are those with: large geographic ranges, large body sizes, large home ranges, generalist habitat requirements, and high natural abundances [6], [32], [39], [40].

It is urgent to document patterns of phyllostomid response in Netropical dry forests as disturbed TDF areas continue increasing in extension and importance [14]. Phyllostomid bats constitute an adequate model for addressing the faunal response to TDF disturbance as they: 1) show specialized requirements for food, roosting sites and habitat selection that make them useful indicators of habitat change [41], 2) include the vast majority of Neotropical nectarivores and frugivores, which play critical roles in ecosystem functioning [12], [42], [43] and 3) constitute the most diversified Neotropical bat family, both in taxonomic and functional terms [42].

The main goal of our study was to identify and explain potential patterns of phyllostomid bat occurrence in patches of TDFs representing different degrees of vegetation development (successional stages), which were defined by their time since abandonment by farmers. These patches represent an important element in natural landscapes affected by anthropogenic activities such as cattle raising and agriculture. In order to identify any potential pattern we documented phyllostomid response in three Neotropical regions: Mexico, Venezuela and Brazil.

First, due to the marked seasonality in the precipitation regime that characterized the TDFs, we documented the seasonal changes on the phyllostomid assemblage attributes (species composition, structure, species richness and abundance) at each sampling site. Then we compared the assemblages (species richness, species and guild composition), ensembles (abundance) and species populations (abundance) occurring in the different successional stages, considering the phyllostomid response to seasonal changes observed in the first analysis. We also analyzed how the variation in vegetation structural complexity could explain the observed bat response.

We expect to find two main patterns at the assemblage level that might further be linked with bat response at the ensemble or population levels. First, seasonal variations in phyllostomid assemblage composition and structure will occur as a consequence of the marked seasonality of TDFs. Specifically, we expect to find an increase in species diversity and bat abundance during the rainy season due to a higher availabity of trophic resources and shelter [20], [31], [32]. Second, bat assemblages associated with different successional stages will also differ in terms of their composition and structure with greater differences occurring between assemblages associated with preserved forests and assemblages associated with patches of secondary vegetation. This difference could mainly be determined by bats of the Phyllostominae subfamily, which are tightly associated with preserved forest [35], [38], [41]. In this sense, it is expected that the region with the highest percentage of species belonging to the Phyllostominae subfamily will show the greatest difference among such assemblages.

## Methods

### 1 Ethics Statement

Bat captures and handling were in accordance with the laws of the Mexican, Venezuelan and Brazilian governments. In Mexico, we have the authorization of the Oficina de Fauna Silvestre (SGPA/DGVS Permit 3644 to KES). This study was also approved by the Secretaría de Medio Ambiente y Recursos Naturales (SEMARNAT), and the Consejo Nacional de Ciencia y Tecnología (CONACYT) from Mexico (Projects 2002-C01-0597 and CB-2005-51043). In Venezuela, we obtained the authorization of the Oficina Administrativa de Permisiones del MINAMB (Licencia de Caza con Fines Científicos 5136 to JAG). In Brazil, we have authorization from the Instituto Brasileiro do Meio Ambiente e dos Recursos Naturais Renováveis - IBAMA (Permit numbers 14654-1 and 14654-2) and from the Instituto Estadual de Florestas (IEF-MG).

### 2 Study Area and Sampling Sites

The study was performed in three regions: 1) the central western coast of Mexico, in and surrounding the Chamela-Cuixmala Biosphere Reserve (hereafter Mexico), wich is located in the state of Jalisco (19°22′–19°35′N, 104°56′–105°03′W) [44]; 2) the west-central portion of the Venezuelan Llanos, in Unidad Productiva Socialista Agropecuaria-Piñero (hereafter Venezuela), an extensive area (∼74,000 ha) located in the state of Cojedes (8°40′–9°00′N, 68°00′–68°18′W) [45]; and 3) the São Francisco River valley in Brazil, in and surrounding the Mata Seca State Park (hereafter Brazil), located in the state of Minas Gerais (14°48′–14°56′S, 43°55′–44°04′W) [46].

These regions, with a marked seasonal rainfall pattern, delineate a gradient of precipitation. In Venezuela, average annual precipitation is 1469 mm, with 86% of the rainfall occurring between May–October [47]. In Brazil, average annual precipitation is 818±242 (SD) mm, with most of the rainfall occurring during November–April [48]. In Mexico, average annual precipitation is 763±258 (SD) mm, with most of the rainfall occurring during June–October [32]. Average annual temperature of the three regions is approximately 25°C [44], [47], [49].

In general, the landscape of the three regions consists of a mosaic of interdigitated forests and open areas where vegetation types are defined based on interactions of elevation, substrate and hydrology, being water availability the most relevant source of environmental heterogeneity for plant establishment and growth [50], [51]. Tropical dry forest (sensu Holdridge) constitutes the predominant vegetation type in these regions, being mostly associated with rolling hills in Mexico, a combination of flatlands and hills in Venezuela and with flat and nutrient–rich soils in Brazil [52]–[54]. In all three regions, small areas of tropical semi-deciduous forest occur along permanent rivers and temporary creeks.

Agriculture and cattle raising are the most important economic activities in the three regions, but tourism is also important in Mexico and Venezuela. As a consequence of these human activities, large areas of these landscapes are covered by cattle ranches, agricultural fields and patches of secondary vegetation representing different successional stages.

In each region we selected twelve sampling sites representing four successional stages (three sampling sites per successional stage), defined in terms of their time since abandonment by farmers (time under natural regeneration process): (1) pastures or initial stages, from P1 to P3, are sites used for cattle raising until the beginning of this study, (2) early successional stages, from E1 to E3, are sites from 3 to 5 years old, (3) intermediate successional stages, from I1 to I3, are sites from 8 to 12 years old in Mexico, 10 to 15 years old in Venezuela and 18 to 25 years old in Brazil, and (4) late successional stages, from L1 to L3, are sites of at least 50 years old. The sampling sites were selected based on the time elapsed since last major disturbance (i.e. cutting, clearing and fire). This information was directly obtained from interviews with farmers and people working in the protected areas. Only sites that had been completely abandoned for an identified period of time were included. Most of the sites were abandoned when economic restrictions limited the farmer’s capacity to use them as agricultural or pasture fields.

Other key characteristics for selecting each sampling site include: (1) their low slope (<25°), allowing the nets to be erected, (2) their distribution around the preserved forest (in order to generate a research design reasonably balanced), (3) their distance from the preserved forest, (4) their accessibility through trails and (5) their availability in each region. The distance from sites to the the preserved forest range from 1000 to 5000 m in Mexico, from 600 to 7000 m in Venezuela, and from 800 to 4000 m in Brazil. The distance among sampling sites range from 500 to 26000 m in Mexico, from 70 to 16650 m in Venezuela, and from 840 to 13500 m in Brazil.

In general, pastures are dominated by non-native grasses and a few shrubs and treelets left standing for fencing and cattle shading. The early successional stage is characterized by an increasing presence of shrubs and the permanence of the non-native grasses and treelets. The height of the 10 tallest woody plants in this stage ranges from 4–7 m in Mexico, 12–16 m in Venezuela, and 5–7 m in Brazil. In the intermediate successional stage, the vegetation shows increments in height, basal area and species number with respect to the early stage. The height of the 10 tallest woody plants here ranges from 10–13 m in Mexico, 7–12 m in Venezuela, and 14–15 in Brazil. Finally, the late successional sites present, on average, the most structurally complex vegetation and the highest species richness [48], [55]. These forests have not suffered any significant human impact for at least 50 years and are now protected at the state or national level. The height of the 10 tallest woody plants in this stage ranges from 9–10 m in Mexico to 20–22 m in Venezuela and 20–23 m in Brazil. The lowest tree height observed in Mexico could be a consequence of the precipitation regime (the longest and driest dry season) and of the topography where the tropical dry forest occurs in this region. These factors determine the soil water availability, which is one of the most relevant factors limiting plant growth in tropical dry forest [51]. The dry forest associated with hills, as is the case of Mexico, could experience lower soil water content than forests located in flat areas due to the elevated evaporative demands associated with a higher insolation rate [56] thus resulting in lower tree growth [51].

### 3 Bat Sampling

Bat sampling was carried out using 2.6 m high mist nets set at ground level. In Mexico, we used a set of five mist nets– two 6-m, two 9-m and one 12-m long; the total sampling area was 109 m^2^ and the study period covered from June 2004 to August 2006. In Venezuela and Brazil we employed a set of ten 12-m mist nets; the total sampling area was 312 m^2^ and the study period spanned from July 2007 to April 2009 and from March 2007 to August 2009, respectively.

Mist nets were located crossing natural or artificial corridors (small corridors of <1 m wide were cleared with a machete) representing potential flyways for bats. Distance among nets was never shorter than 30 m, and sampling was always performed during the first 5 hours after sunset, a period of time that coincides with the foraging peak for most phyllostomid bats [57]. In order to avoid variation in capture success, we sampled during non-rainy, non-windy and moonless nights. In addition, to avoid biases due to trap-shy behavior of bats, each site was sampled a single night during each sampling period and the order in which each site was sampled was randomized [58].

Sampling efforts encompassed the rainy (RS) and dry (DS) seasons. Seasons were defined in accordance with the precipitation regime during the study period. The RS was considered to begin one month after the first rain, while the DS was considered to begin one month after the last rain [59]. In Mexico, sites were sampled 5±1.6 (SD) nights during the RS and 9±1.6 (SD) nights during the DS. In Venezuela, sites were sampled 4±0.8 (SD) nights during the RS and 4±0.3 (SD) nights during the DS. Finally, in Brazil, sites were sampled 4±0.8 (SD) nights during the RS and 7±1.1 (SD) nights during the DS.

During sampling, nets were checked every 30 min and all captured bats were stored in cloth bags. Pregnant females and juveniles were processed first and released. Bat species were identified based on the field guides of Linares [60], [61], Timm and Laval [62] and Medellín et al. [63]. We also considered the study of Nogueira [64] carried out in Jaíba, Brazil. A small set of voucher specimens (1–5 individuals) belonging to species with uncertain identification were sacrificed and preserved in 70% ethanol for posterior analysis. With the exception of juveniles and non-healthy individuals, all captured bats were marked on their forearm with a numbered aluminum band in Mexico and Brazil. In Venezuela bats were marked temporarily by cutting a small piece of hair from the center of their back. This allowed recaptures during any single sampling period to be identified.

Taxonomic designation of bat species follows Simmons [65]. We assigned bat species to broad guilds based on Kalko et al. [66], Timm and Laval [62], Castro-Arellano et al. [67] and Reis et al. [68]. The recognized guilds for phyllostomids were: nectarivores, frugivores, gleaning insectivores, omnivores, carnivores and sanguivores.

### 4 Vegetation Structural Complexity

Vegetation structure of each sampling site was characterized within a 0.10 ha (20 x 50 m) plot, considering all woody plants with a diameter at breast height (DBH) ≥5 cm [90]. The vegetation attributes measured were number of individuals (NI), number of species (NS), and total basal area (BA).

In order to obtain a continuous synthetic variable summarizing sampling site variation in terms of vegetation structural complexity, we performed a principal component analysis (PCA) considering NI, NS and BA. The new variable (axis 1 scores, Fig. S1) was used as an explanatory variable for evaluating phyllostomid response to changes in vegetation structure.

The total variation explained by PCAs axis 1 was 93%, 86% and 94% for Mexico, Venezuela and Brazil respectively. All vegetation parameters considered in the PCAs were positively correlated with axis 1. The corresponding eigenvector values were: NI = 0.58, NS = 0.58 and BA = 0.57 for Mexico; NI = 0.57, NS = 0.58 and BA = 0.58 for Venezuela, and NI = 0.58, NS = 0.57 and BA = 0.58 for Brazil. All analyses were performed in R (v.2.11.1) [69], using the “prcomp” function available in the stats package.

### 5 Data Analysis

Sampling completeness per site was evaluated through the percentage of species captured in each site in relation to the site’s estimated species richness. Each season was considered separately. Species richness was estimated using the first-order jackknife estimator in EstimateS (v.8.2) [70], which is based on incidence data considering the number of species occurring in a single sample [71]. We selected this estimator because it produces a low-bias estimation of species richness even at small sample sizes (<100 individuals per site, Colwell and Coddington [72]). In accordance with Moreno and Halffert [73], we considered 90% of completeness a sufficient level of sampling efficiency.

#### 5.1 Seasonal variation in phyllostomid assemblages

We analyzed bat assemblage seasonal variations in terms of species composition, structure, species richness and abundance. For this purpose, in each site we compared RS versus DS phyllostomid assemblages.

As a first step, in order to graphically represent assemblage attributes per site and per season, we built rank-abundance (dominance-diversity) graphs following Feinsinger [74]. We graphed the “log_10_ pi” (pi being the proportion of individuals of a given species relative to all captured individuals) versus the species ranked from left to right according to their relative abundance. This method allows visualizing some assemblage attributes such as species richness (number of points), evenness (slope), number of rare species (tail of the curve) and species relative abundance (order of the species in the graph). Indeed, plotting the “log_10_ pi” instead of the total number of captured individuals per species, facilitates the comparison between curves differing in their total number of individuals.

In order to compare the species composition of RS and DS assemblages, we carried out an X^2^ randomization test in the EcoSim software (v.7.72) [75], using null models based on 1000 random rearrangements. To compare assemblage structure (species rank distribution) between seasons, we performed the Kolmogorov-Smirnov test [76] in R (v.2.11.1) [69]. Species richness was compared by using the first-order jackknife estimator; the lack of overlapping of its 95% confidence intervals was considered an indicator of significant differences between RS and DS assemblages [77]. Finally, to compare bat abundance, we used the capture rate (individuals/night) as an indicator of local abundance in each season. Then, we performed a Monte Carlo permutation test for comparing the abundance of bats during the RS versus the DS. For this purpose, we used the R package “coin” [78] generating the reference distribution with 10,000 rearrangements. The Monte Carlo method is useful to analyze unbalanced data as well as variables that are not normally distributed.

#### 5.2 Phyllostomid occurrence in different successional stages

Phyllostomid occurrence in the different sucesional stages was evaluated at the species, ensemble and assemblage-level. The RS and DS captures were also separately analyzed for this purpose. At the species and ensemble levels we used the capture rate (individuals/night) per site and per season as an indicator of local abundance. Only species and ensembles with at least 10 captured individuals in each season were analyzed. At the assemblage-level, we used as response variables the species richness estimated by the first-order jackknife estimator and four new synthetic variables reflecting the dissimilarities among the assemblages in terms of species (variables 1 and 2) and guild composition (variables 3 and 4). These synthetic variables were obtained by mapping, through a non-metric multidimensional scaling ordination (NMDS) [79], the dissimilarities among the assemblages (one ordination for species and one ordination for guild). The sampling site scores from axis 1 and 2 of the two resulting bidimensional ordinations were then used as the synthetic variables. Both ordinations were based on Bray-Curtis similarity matrices of species and guilds [71].

The Bray-Curtis measure of distance allows the comparison of assemblages in terms of species/guilds presence and abundance [71]. For calculation of the Bray-Curtis coefficients, the abundance data corresponding to all sites were standardized to the same abundance by dividing each cell abundance (species or guild abundance) by the total site abundance (total captured individuals in the site). In this way, we compared sampling sites, in terms of species or guild proportions, ensuring that differences in total abundance among sites would not influence the results [80]. All phyllostomids, including those scarcely represented (1 or 2 individuals), were considered in the analysis. Recaptures were not included. The degree of relation between each species/guild and the sampling sites was mapped after computing the weighted average scores of species/guild for the resulting ordination configuration [81]. Although we did not identify long-term recaptures in Venezuela because bats were not permanently marked, we are confidant this did not significantly affect our results because of the very low recapture rate observed during sampling (ranging between 1.2 to 4% in Mexico and Brazil, respectively). Most of the recaptured individuals in both regions belonged to the species *Desmodus rotundus*.

We assessed the causal-explanatory relationships between all response variables and two explanatory variables, successional stage and vegetation structural complexity (scores of the PCA axis 1), through a hierarchical partitioning analysis [82]. This is a regression technique where all possible GLMs combining the explanatory variables are jointly considered in order to obtain a measure of the independent effect of each explanatory variable. For this purpose, the increment that a particular explanatory variable generates in the model fitting is estimated by averaging the variable influence over the whole model [82]. This procedure alleviates problems of multicollinearity between the explanatory variables [82]. The significance (α = 0.05) of the relationships between explanatory and response variables was evaluated through the randomization test suggested by Mac Nally [83].

Abundance data were modeled by using a Poisson error distribution with the log link function, while the response variables generated through NMDS were modeled using the Gaussian error distribution with the identity link function. When needed, the response variables were transformed in order to fit normality. In all cases the goodness of fit was based on log-likelihood.

The pasture sites of Mexico had to be discarded from the analysis because of their low capture rates. The site L2 of Mexico was not considered in the hypothesis test evaluating the variation in phyllostomid assemblages in terms of species and guild composition, as no individuals were captured at this site during the DS. Zero values in capture data result in a meaningless value of the Bray-Curtis coefficient. The site P1 of Venezuela also was excluded from the analysis due to small sample size during the DS. Finally, the site L3 of Brazil was not considered in the analysis due to logistical problems in vegetation sampling.

All statistical analyses described in this section were performed in R (v.2.11.1) [69]. We used the vegan package for the Bray-Curtis coefficient calculation and NMDS ordinations (function “metaMDS”) [84]. Hierarchical partitioning analysis was performed using the “hier.part” package [85].

## Results

One hundred and forty-two sampling nights were performed in Mexico, 63 during the RS and 79 during the DS, leading to the capture of 606 phyllostomid bats representing 15 species, 11 genera, 5 subfamilies and 4 broad guilds (Table S1, Result S1). Nine of the 15 species were captured in both seasons while 6 were exclusively captured during the RS: *Micronycteris microtis*, *Choeroniscus godmani*, *Musonycteris harrisoni*, *Centurio senex*, *Chiroderma salvini* and *Carollia* sp. In Venezuela we conducted 94 sampling nights, 50 in the RS and 44 in the DS, allowing the capture of 996 phyllostomid individuals representing 30 species, 17 genera, 5 subfamilies and 6 broad guilds (Table S1, Result S1). In Venezuela most of the species were captured in both seasons, with the exception of 5 species exclusively captured during the DS (*Micronycteris hirsuta*, *Mimon bennettii*, *Mimon crenulatum*, *P. stenops*, *Tonatia saurophila*) and 3 species exclusively captured during the RS (*A. lituratus*, *M. microtis*, *Vampyrum spectrum*). Finally, in Brazil we performed 130 sampling nights, 46 during the RS and 84 during the DS, capturing 808 phyllostomid individuals classified in 19 species, 15 genera, 5 subfamilies and 6 broad guilds (Result S1). Due to dubious differentiation between *Carollia perspicillata* and *Carollia brevicauda*, we decided to group both species into the single taxon *Carollia* spp. This taxon was used as a unit during analyses. Most of the species were captured in both seasons, excluding two species exclusively captured in the RS (*Chiroderma villosum*, *Mimon bennetti*) and one species captured in the DS (*Sturnira lilium*).

Although the same 5 subfamilies were represented in the three study regions, in Mexico the best represented subfamily was Stenodermatinae, whereas in Venezuela and Brazil the best represented subfamily was Phyllostominae. (Fig. S2). Venezuela and Brazil also presented the highest similarity regarding guild composition. In these regions, six broad guilds were represented among the captured species: omnivores, carnivores, gleaning insectivores, nectarivores, frugivores and sanguivores, whereas only the last four guilds were observed in Mexico. The frugivore ensemble was the most speciose in all regions, followed by the gleaning insectivores in Venezuela and Brazil and the nectarivores in Mexico.

The sampling effort used in the three regions was considered sufficient to characterize the phyllostomid assemblages occurring in each sampling site. Completeness reached 90% in all cases, ranging from 90 to 100% in Mexico, 95 to 100% in Venezuela and 91 to 100% in Brazil (Table S1). Nevertheless, a high inter-annual rate of species turnover was observed in several sampling sites. In some cases a complete change of species occurred from one year to another (Table S2).

### 1 Seasonal Variation in Phyllostomid Assemblages

**Figure 1:**
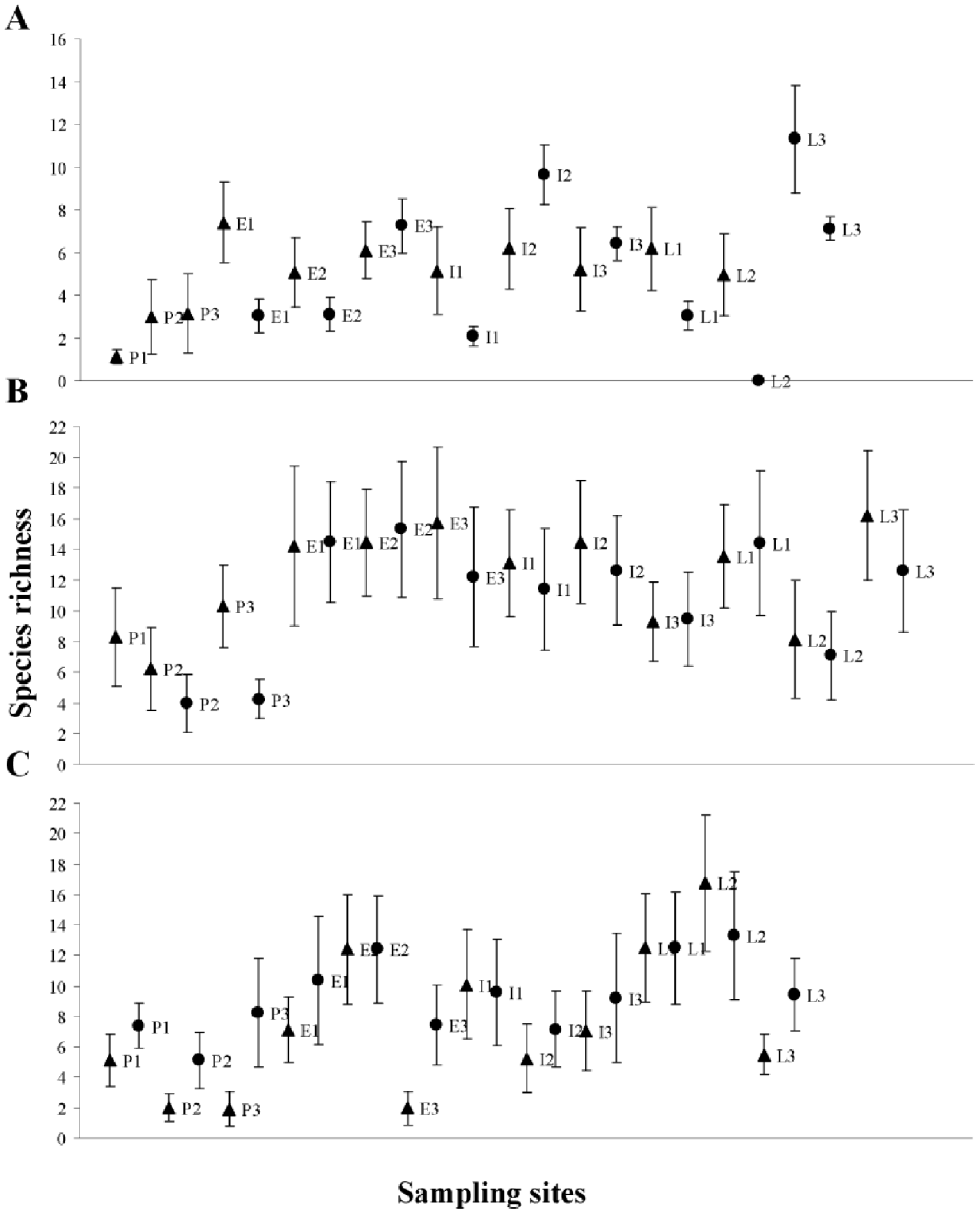
Species richness estimated with the first-order jackknife estimator, per site and per season. Study regions: Chamela Cuixmala Biosphere Reserve in Mexico (A), Unidad de Producción Socialista Agropecuaria Piñero in Venezuela (B), and Mata Seca State Park in Brazil (C). Sampling sites representing different successional stages are: pastures (from P1 to P3), early (from E1 to E3), intermediate (from I1 to I3) and late stage (from L1 to L3). Seasons: rainy season (triangles) and dry season (circles). Error bars represent the ±95% confidence intervals.

**Figure 2:**
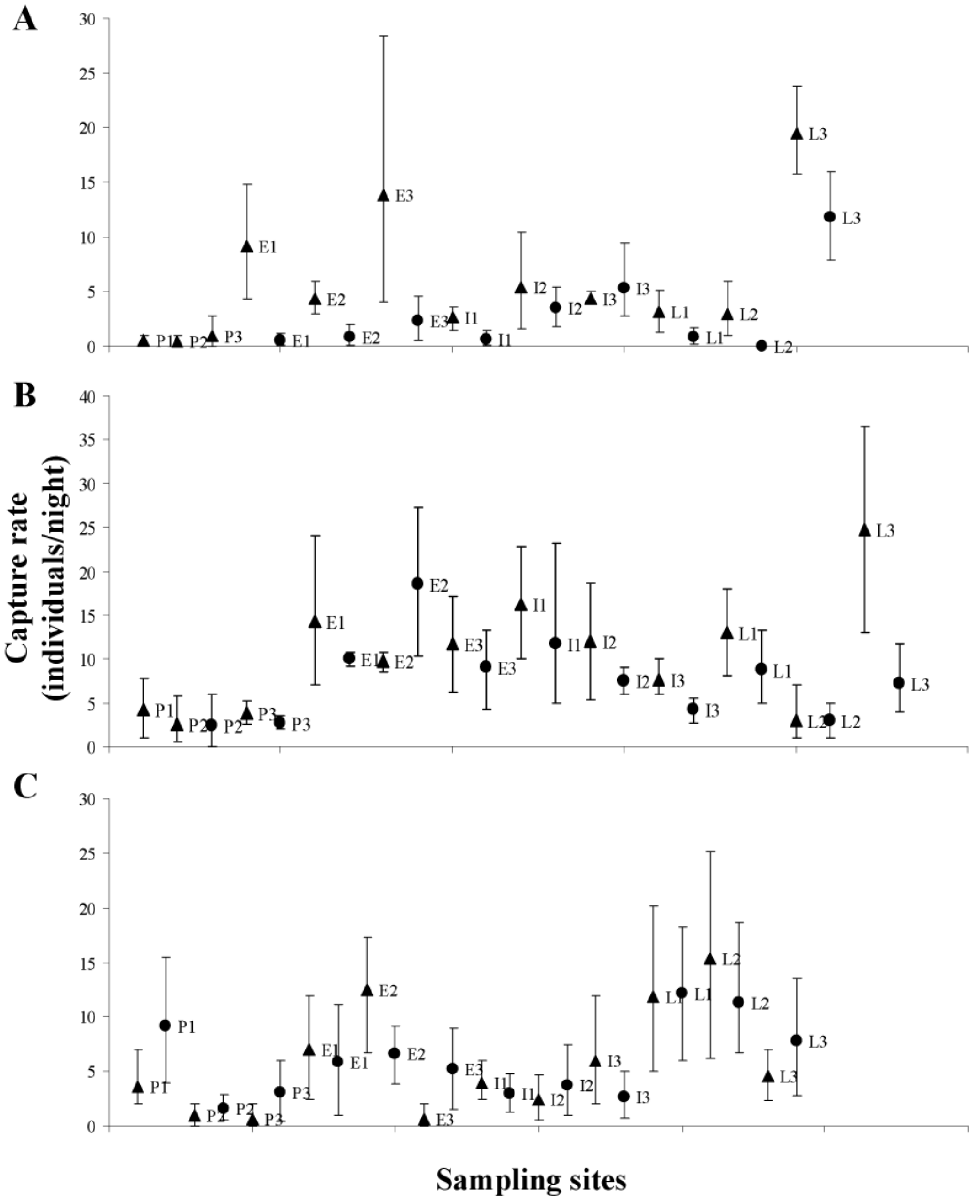
Capture rate (individuals/night) of phyllostomid bats per site and per season. Sites and stages described in Fig. 1.

In general, most of the seasonal variations in phyllostomid assemblages were observed regarding species composition, species richness and bat abundance (Table 1). In just a few sites, we detected seasonal fluctuations regarding the assemblage structure. Specifically in Mexico, the main seasonal variations in phyllostomid assemblages were observed in terms of species richness and bat abundance. For example, during the DS, there was a significant reduction of species richness in 5 sampling sites (Fig. 1), as well as a significant reduction in bat abundance in 6 sites (Fig. 1, Fig. 2, Table 1). Significant variations in species composition occurred only in the site E3. In most of the sites, the most abundant species during the RS were also the most abundant species during the DS (Result S1). Nevertheless, we identified species exclusively represented in a single season (mainly the RS) for most of the sites. Most assemblages were dominated by *Artibeus jamaicensis* and *Glossophaga soricina* during the RS and by *A. jamaicensis* during the DS (Result S1).

**Table 1:** Summary of the tests evaluating seasonal variation at the assemblage level.

	Assemblage-level parameters			
	Composition	Abundance	Richness	Structure
Site	Z-score;p-value	Z-score;p-value	Symbol	D;p-value
**Mexico**				
E1	−0.599; 0.698	−**2.661; <0.001**	<	0.714; 0.234
E2	−0.463; 0.666	−**2.307; 0.026**	=	0.800; 0.181
E3	**4.734; 0.001**	−**1.864; 0.024**	=	0.262; 0.980
I1	1.243; 0.104	−**2.375; 0.020**	<	0.800; 0.320
I2	−0.902; 0.836	−0.856; 0.460	>	0.222; 0.994
I3	−0.351; 0.583	0.318; 0.967	=	0.500; 0.503
L1	−1.449; 0.981	−***1.940; 0.062***	<	0.500; 0.699
L2	–	−**2.121; 0.013**	<	–
L3	−0.579; 0.696	−**2.072; 0.034**	<	0.442; 0.375
**Venezuela**				
P2	**1.990; 0.040**	−0.112; 1.000	=	0.667; 0.237
P3	**2.625; 0.014**	−1.018; 0.543	<	***0.800; 0.052***
E1	**4.777; <0.001**	−1.017; 0.514	=	0.429; 0.152
E2	**3.903; <0.001**	1.535; 0.143	=	0.400; 0.197
E3	**1.848; 0.043**	−0.602; 0.610	=	0.333; 0.449
I1	**4.800; <0.001**	−0.693; 0.571	=	0.357; 0.435
I2	1.281; 0.112	−0.953; 0.405	=	0.441; 0.163
I3	**2.741; 0.014**	−***1.791; 0.086***	=	0.444; 0.336
L1	***1.736; 0.052***	−1.116; 0.257	=	0.308; 0.546
L2	−0.009; 0.460	0.000; 1.000	=	0.214; 0.996
L3	**6.488; <0.001**	−***1.814; 0.055***	=	**0.563; 0.026**
**Brazil**				
P1	0.942; 0.155	1.054; 0.367	=	0.500; 0.425
P2	−1.439; 0.984	0.587; 0.709	>	0.750; 0.441
P3	0.096; 0.402	0.943; 0.425	>	0.875; 0.173
E1	−1.367; 0.947	−0.272; 0.779	=	0.400; 0.525
E2	−1.720; 0.974	−***1.732; 0.078***	=	0.250; 0.848
E3	−0.619; 0.729	1.460; 0.257	>	***1.000; 0.089***
I1	−0.968; 0.833	−0.659; 0.527	=	0.444; 0.307
I2	***1.528; 0.074***	0.472; 0.788	=	0.571; 0.297
I3	0.275; 0.333	−1.216; 0.283	=	***0.667; 0.060***
L1	**7.237; <0.001**	0.067; 0.980	=	0.333; 0.518
L2	0.405; 0.330	−0.672; 0.544	=	0.332; 0.409
L3	0.534; 0.258	0.781; 0.507	>	0.556; 0.274

Study sites are the same as described in Fig. 1. For tests based on randomizations (composition and abundance) the standardized effect size is provided (Z = (Observed value – Expected value)/StDev of expected values). The Z-score quantifies, in units of standard deviation, the position of the observed metric within the simulated distribution. Significant relationships (p-value ≤0.05) appear in bold and marginally significant relationships (0.05<p-value ≤0.10) appear in italic and bold. Significant differences regarding species composition are indicated when the Z-score is significantly large (p (observed ≥ expected)), which means that the observed data fit the expected values significantly worse than the simulated data. Species richness: no differences between season ( = ), significant reduction during the dry season (<), and significant increase during the dry season (>). Structure: result of the Kolmogorov-Smirnov test evaluating seasonal changes in bat assemblages regarding their structure (species rank distribution). Detailed information about these analyses are presented in the method section.

In contrast to our observations in Mexico, in Venezuela the main seasonal variations in phyllostomid assemblages occurred in terms of species composition (Table 1, Result S1). Species exclusively represented in a single season occurred in all sites; the highest numbers of these species were registered during the RS. Most of the assemblages were dominated by *Phyllostomus elongatus* during the RS (Result S1) and by a diverse arrangement of species (*S. lilium*, *Glossophaga longirostris*, *Uroderma bilobatum*, *Platyrrhinus vittatus* and *P. elongatus*) during the DS. Species richness showed seasonal variation only in the site P3 (Fig. 1), whereas the assemblage structure showed seasonal variation only in the site L3. Finally, we did not detect any significant seasonal variation in bat abundance, but in sites I3 and L3 we found a tendency toward lower abundance during the DS (Fig. 2, Table 1).

In general, for most of the Brazilian sites, we did not detect marked seasonal variation in assemblage attributes. A significant seasonal variation in species composition was observed only in a single site (L1, Table 1). In most sampling sites, the most abundant species during the RS were among the most abundant ones during the DS (Result S1). In addition, we observed seasonal fluctuations in species richness in four of the sampling sites (P2, P3, E3, L3), in which a higher number of species occurred during the DS (Fig. 1). Finally, no seasonal variations in bat abundance and assemblage structure were registered at any site in this region (Fig. 1, Table 1).

### 2 Phyllostomid Occurrence in Different Successional Stages

#### 2.1 Ensemble and species level response

We found different responses among the regions in the occurrence of frugivorous bats in the different successional stages, specifically between Venezuela versus Mexico and Brazil. In Venezuela, frugivore abundance (Table 2) was significantly higher in the early successional stage during the DS. This stage also showed higher abundance of the frugivorous species *Uroderma bilobatum* (during the RS and DS) and *U. magnirostrum* (during the DS) (Result S2). Nevertheless, variations of some frugivorous populations in this region were more tightly associated with variation in vegetation structural complexity than to successional stage (Table 2). This is the case of *A. jamaicensis*, *C. brevicauda* and *P. vittatus*, which during the RS, reached their highest abundance in sites with higher structural complexity (Table 2). In contrast, *Sturnira lilium* abundance was higher in sites with lower structural complexity during the DS. In the case of Mexico and Brazil, frugivore abundance did not show significant differences among successional stages, although highest abundances were always found in late successional sites (Table S3).

**Table 2:** Percentage of variation in the population, ensemble and assemblage-level parameters associated with the variation of the explanatory variable.

				Explanatory variables	
Response variable	Guild	n	*R^2^_dev_*	S_stage_	V_struct_
**Mexico**					
***RS***					
* Glossophaga soricina*	N	9	0.795	**81.649**	18.351 (−)
Nectarivores		9	0.723	**79.907**	20.093 (−)
Species SC_2_		8	0.809	**73.786**	26.214 (−)
Guild SC_2_		8	0.795	**76.667**	23.333
***DS***					
**Venezuela**					
***RS***					
* Artibeus jamaicensis*	F	11	0.903	8.789	**91.211**
* Carollia brevicauda*	F	11	0.662	7.631	**92.369**
* Platyrrhinus vittatus*	F	11	0.483	2.264	**97.736**
* Uroderma bilobatum*	F	11	0.760	**73.936**	26.064
* Desmodus rotundus*	S	11	0.690	**99.086**	0.914
Omnivores		11	0.729	**98.638**	1.362
Species SC_1_		11	0.945	2.363	**97.636**
Guild SC_2_		11	0.858	**58.686**	**41.314** (−)
Jack1		11	0.484	**90.031**	9.969
***DS***					
* Sturnira lilium*	F	11	0.868	35.694	**64.306** (−)
* Uroderma bilobatum*	F	11	0.792	**98.841**	1.159
* Uroderma magnirostrum*	F	11	0.847	**94.272**	5.728 (−)
* Phyllostomus elongatus*	O	11	0.356	**96.636**	3.364
Frugivores		11	0.615	**99.324**	0.676
Gleaning insectivores		11	0.614	2.151	**97.849**
Omnivores		11	0.355	**96.662**	3.338
Species SC_1_		11	0.748	5.666	**94.334**
Guild SC_2_		11	0.438	8.227	**91.773**
Jack1		11	0.769	**69.956**	30.043
**Brazil**					
***RS***					
* Desmodus rotundus*	S	11	0.603	1.066	**98.934**
Sanguivores		11	0.631	1.165	**98.835**
Jack1		11	0.665	1.763	**98.237**
***DS***					
* Glossophaga soricina*	N	11	0.554	**84.394**	15.606
Nectarivores		11	0.375	**97.417**	2.583
Jack1		11	0.679	6.876	**93.124**

Study sites are the same as described in Fig. 1. Seasons: rainy season (RS), and dry season (DS). Parameters at population-level: capture rate (individuals/night) as an indicator of local abundance of the species. Parameters at ensemble-level: capture rate (individuals/night) as an indicator of local abundance of the guild. Parameters at assemblage-level: scores of the first and second ordination axis reflecting assemblages’ dissimilarities in species composition (Species SC_1_ and Species SC_2_, respectively); scores of the second ordination axis reflecting assemblages’ dissimilarities in guild composition (Guild SC_2_), and species richness estimated by using the first-order jackknife estimator (Jack1). Explanatory variables: successional stage (S_stage_) and scores of the first ordination axis reflecting sampling sites’ dissimilarities in vegetation structural complexity (V_struct_). n: number of sampling sites. *R^2^_dev_* is the fraction of the total deviance explained by a model considering all explanatory variables when the Poisson error distribution was used and *R^2^* when the normal error distribution was used. Significant relationships according to the randomization test appear in bold. Negative relationships are shown in parentheses. Only the parameters that were significantly associated with some explanatory variable are shown in this table. See Table S3 for all the results.

Regarding nectarivore abundance, the response in Mexico and Brazil was similar as well. In both regions, nectarivore abundance was significantly different among successional stages (Table 2), being higher in the early successional sites at both the ensemble and species level (i.e. *G. soricina,* Result S2). These differences occurred during the RS in Mexico and during the DS in Brazil. Nectarivore abundance did not differ among successional stages in Venezuela.

Gleaning insectivore abundance, evaluated only in Venezuela and Brazil, did not differ among successional stages (Table S3). In Venezuela some gleaning insectivorous species tended to be more represented in the early and intermediate sites (i.e. *Lophostoma brasiliense*, *Trachops cirrhosus*, *Phylloderma stenops*). However, the variation in their abundance was positively related to variations in vegetation structural complexity (during the DS) (Table 2). The unique individual representing this guild in Mexico was captured in a late successional site during the RS (Result S1).

Omnivorous bats were only found in Venezuela and Brazil and they showed different responses. In Venezuela, their occurrence was significantly different among successional stages (Table 2) with the highest abundance achieved in the early and intermediate sites at both the ensemble and species level (*P. elongatus).* Higher total abundance in these successional stages occurred during both seasons (Result S2). In Brazil, in contrast, omnivore abundance did not differ among successional stages (Table S3).

The abundance of *D. rotundus,* the only sanguivorous species found in all three regions, showed different responses among the regions (Table 2). In Mexico, the variation in their abundance was not associated with any explanatory variable. In Venezuela its abundance differed significantly among successional stages during the RS, being greatest in early successional sites (Result S2). Finally, in Brazil, *D. rotundus* abundance, as well as the abundance of all sanguivorous bats (including *Diphylla ecaudata*) were significantly higher in sites with higher vegetation structural complexity (during the DS, Table 2).

#### 2.2 Assemblage-level response

The variation among phyllostomid assemblages occurring in the different successional stages, in terms of species and guild composition, was different for each region (Table 2). In Mexico, most of the variation was explained by successional stage. Late successional assemblages significantly differed from early and intermediate assemblages in terms of species and guild composition during the RS (Result S2 and S3). However, during the DS, differences in species composition were registered between early and intermediate-late succession (Result S2 and S4). In the case of Venezuela, variations of species and guild composition were explained by variations in vegetation structural complexity among sites in both seasons (Table 2). Nevertheless, similar to Mexico during the RS, differences in guild composition were also explained by successional stage, with late successional assemblages differing significantly from early and intermediate assemblages (Result S2 and S3). Finally, in Brazil, variations in terms of species and guild composition were not significantly related to successional stage nor vegetation structural complexity (Table S3, Result S3and S4).

Assemblage variation in terms of species richness also was explained differently for each study region. Neither the type of successional stage, nor variations in vegetation structural complexity significantly explained the differences in species richness among the Mexican sites (Table S3). In Venezuela, however, early successional sites showed the highest species richness in both seasons (Table 2, Result S2). Finally, in Brazil, variation in assemblage species richness was positively and significantly associated with variations in vegetation structural complexity during both seasons (Table 2).

## Discussion

### 1 Seasonal Variation in Phyllostomid Assemblages

As expected, we found evidence of seasonal variation in phyllostomid assemblage attributes in the three study regions. These variations occurred both at the regional and sampling site levels. In all regions we registered species that were exclusively captured during a single season. In Mexico and Venezuela, most of these species were captured during the RS, whereas in Brazil they were captured during the DS.

The observed seasonal variations in phyllostomid assemblages could be a consequence of the marked seasonality in the precipitation regime of these TDFs [47], [62], [63]. The seasonal change in water availability provokes distinctive plant phenological patterns, changes in the primary productivity [5], [86] and consequently, seasonal variations in vegetation structural complexity and bat resource availability (i.e. food and roosts) [17], [20]. Indeed, in the study sites of Mexico and Brazil, significant seasonal variation in plant phenology (leafing, flowering and fruiting), is found between successional stages [87]–[89]. In addition, seasonal fluctuations in the chiropterophilic and chiropterochoric resources, as well as in the amount of insects, are found in the three regions (per. obs) [20], [86]. Chiropterophilic resources peak during the mid-dry season and at the beginning of the RS, whereas the highest number of trees with chiropterochoric fruits occurrs during the RS (per. obs) [20], [88], [90]. These variations could also explain the seasonal differences in the association between phyllostomids and successional stages or between phyllostomids and vegetation structural complexity.

Bats cope with seasonal variations in key resources through behavioral changes in diet breadth, type of food, habitat use and defense of feeding areas, as well as through migration [23]–[26]. For example, in Mexico [26] and Venezuela [90], insects become an important source of protein for nectarivorous and frugivorous bats during the DS, being more important as a source of proteins (51–75%) than plants. Moreover, seasonal changes in bat foraging behavior and home range size can be caused by the seasonal breeding pattern that is characteristic of a high percentage of Neotropical phyllostomids [33]. These behavioral changes can be reflected in the observed seasonal variations of bat assemblages in terms of species richness, composition and abundance.

The nature and intensity of seasonal variations in bat assemblages differed among the regions. The most marked variations were detected in Mexico, where species richness and bat abundance of most assemblages significantly decreased during the DS. In Venezuela and Brazil, these parameters did not significantly change between seasons in most sites. Observed differences among the regions are probably a consequence of their climatic specificities. Mexico presents the lowest precipitation level (see methods), as well as the lowest amount of available water across the year. The main sources of water around sampling sites consist of seasonally drying water bodies, such as temporary rivers and creeks. In this context, migration has been reported as one of the most important behavioral adaptations of the regional mammals to deal with water and food availability reductions during the DS [91]. As bats are among the mammals with the highest capability of movement, their seasonal migration to resource richer areas would explain their reduction in species richness and abundance during the DS [92]. Latitudinal migration has been well documented in the region for *L. yerbabuenae*, whose migration to northern latitudes is tightly associated with the regional reduction in their food availability during the DS [19]. In contrast, bats inhabiting in Venezuela and Brazil can find permanent sources of water all year-round in the regional rivers and lagoons. In Venezuela Laguna Grande is located from 100 m to 16,500 m from sampling sites and Lagoa da Prata and Rio São Francisco in Brazil are located from 900 m to 8,000 m from sampling sites. The presence of these water bodies might partly explain the absence of significant seasonal changes in the species richness and bat abundance of Venezuela and Brazil assemblages.

Finally, the significant seasonal changes in species composition of the Venezuela assemblages, appear to be a distinctive feature of this region. In 2003, Aguirre et al. [93] report a high species turnover, even among years, for a similar environment in Bolivia. In this study, the availability of the main sources of food significantly determined bats’ permanent membership in the assemblages. Specifically, bats relying on fluctuating sources of food are prone to be absent during certain periods of time, whereas those relying on more permanent sources of food are prone to be continuously present in the assemblages. Indeed, a high inter-annual rate of species turnover also was detected in Mexico and Brazil. This can be a consecuence of the regional inter-annual variations in plant phenology and in others sources of food (i.e insects).

### 2 Phyllostomid Occurrence in Different Successional Stages

We found a high seasonal and regional level of specificity in phyllostomid occurrence across different successional stages. This specificity was related to the variation in environmental conditions and resource availability, as well as to particularities in the phyllostomid response at species and guild levels.

#### 2.1 Ensemble and species level response

The specificity of phyllostomid response to habitat change associated with succession was related to the contrasting ecological requirements of bat species and guilds. For example, frugivore abundance was higher in the early stage at Venezuela, but it did not show a marked difference among successional stages in Mexico or Brazil. The pattern observed in Venezuela, which is the region with the highest average annual precipitation (1469 mm) [47], is indeed more similar to what has been observed in more humid forests [36] where frugivore abundance is higher in early successional stages. Several individuals of chiropterochoric species belonging to the genera *Annona*, *Cecropia* and *Ficus* are found in the Venezuela early successional sites (Nassar et al. unpublished data). Chiropterochoric species are common in early succession of tropical wet and rainy forests [8], [13], [36]. In the Colombian Amazon, for example, individuals from the chiropterochoric genera *Cecropia*, *Miconia*, and *Vismia* accounted for 87% of the stems present in an area abandoned after three years of slash and burn agriculture [12]. Moreover, differences in frugivore responses among regions could be explained by differences in the regions’ environmental characteristics (e.g. precipitation regime) reflected in the species composition of their early successional plant assemblages. In this sense, the absence of this pattern in Mexico and Brazil, two TDFs with a lower average annual precipitation (763 and 818 mm respectively), would be related to an early successional stage dominated by anemochorous plants [32], which do not constitute food resources for frugivorous bats.

Regarding nectarivorous bats, we found a similar response in Mexico and Brazil. In both regions the early successional stage, characterized by the presence of shrubs and some non-native grasses, showed a significantly higher nectarivore abundance at both the ensemble and species level (*G. soricina*). This is likely related to (1) the capacity of the most abundant nectarivores (Mexico: *G. soricina*, and *L. yerbabuenae;* Brazil: *G. soricina*) to forage and exploit resources in areas where vegetation has a simple structure [94], and (2) the greater occurrence of chiropterophilic plants in the early successional stage of both study sites; these plants mainly belong to the genera *Acacia* and *Cordia* [43], [95].

Contrary to our expectations, the occurrence of gleaning insectivores did not differ among successional stages in the two regions in which they were analyzed (Venezuela and Brazil). Gleaning insectivore occurrence appears to be more tightly associated with variations in vegetation structural complexity as found in Venezuela (Table 2). This causal/explanatory relationship may be explained by the guilds’ high specialization for foraging (gleaning insects from leaves or other surfaces in a highly cluttered space) [66] and for roosting (large, shaded leaves, termite nests and hollow trees) [35]. In fact, we found great variations among sites of the same successional stage in terms of vegetation structural complexity (Fig. S1). Moreover, some species thought to be mainly associated with the most advanced sucesional stages were found in earlier stages (in Venezuela: *L. brasiliense*, *T. cirrhosus*, *P. stenops*). In Venezuela, the early successional sites presented a structurally complex vegetation as a consequence of their specific land-use history. When farmers cleared these sites, they left several remnant trees favoring a more complex vegetation structure. In adittion, two of the three sites representing this stage are close to one body of water (100 m far from the Laguna Grande lagoon), which attracts more prey used by bats such as insects, frogs, and other small vertebrates. The combination of these conditions could explain why these species were associated with early successional sites.

Omnivorous and sanguivorous bat occurrence in the different successional stages showed a high variability among regions. In the case of omnivores, this variability may be associated with the high diversity of food resources they use [66], [68] as well as to regional differences in the type and distribution of such resources. A further detailed analysis of omnivore preferences in each region must be performed in order to identify the “drivers” of their distribution. On the other hand, factors explaining the inter-regional variation in sanguivore response to habitat change could be related to the distribution of cattle, a non-native source of food prefered by *D. rotundus* in anthropogenic tropical landscapes. Differences in the distribution and coverage of riparian vegetation around the sampling sites can also be influencing sanguivore occurrence. Riparian vegetation may affect sanguivore distribution in anthropogenic landscapes [94] for the following reasons: (1) it offers roosting sites and can be used as stepping stones when bats search for food in the vegetation matrix where cattle occur, (2) it presents a higher availability of native food sources for sanguivores (medium and large-sized mammals that concentrate their activities in riparian vegetation) when resources are limited outside of the riparian habitat, and (3) farmers concentrate cattle in riparian areas, especially when water availability is low.

#### 2.2 Assemblage-level response

In contrast to our prediction, the region with the highest proportion of Phyllostominae (Venezuela) did not show differences among successional stages in relation to assemblage species and guild composition. On the contrary, the region with the poorest representation of Phyllostominae (Mexico, with just 1 species, *Micronycteris microtis*), presented the highest differentiation in assemblage species and guild composition among successional stages.

Our results probably reflect the specific nature of the factors determining the occurrence of most species in the study regions. In Venezuela for example, variations in assemblage composition are mainly explained by variations in the vegetation structural complexity. This is congruent with our finding that variations in the abundance of gleaning insectivores, the most speciose ensemble, are also explained by changes in the vegetation structural complexity, and not by successional stage. In the same sense, we found that some of the most abundant frugivores in this region also responded to changes in vegetation structural complexity (*A. jamaicensis*, *C. brevicauda*, and *P. vittatus*, Table 2).

In Mexico however, the occurrence of several species appears to be determined by successional stage. This is the case of the three most abundant nectarivores, which occur in higher abundance in the early stage (Table 2, Result S2), as well as of the species exclusively captured in late successional sites (*Carollia* sp., *C. senex*, *C. salvini*, *C. godmani*, *M. microtis*, *M. harrisoni*) [32], [94]. These species together represent 67% of all the species reported in Mexico during our study.

In relation to phyllostomid species richness, no consistent patterns were detected among the study regions. Whereas in Mexico none of the explanatory variables were significantly related to the variations in species richness, in Venezuela this parameter was explained by successional stage and in Brazil it was explained by vegetation structural complexity. These differences among the regions must be due to inter-regional variations in other non-measured factors likely related to attributes of the landscape surrounding the sampling sites.

In the case of Mexico, we detected high intra-successional stage variation in assemblage species richness. This variation was not explained by differences in vegetation structural complexity among sites. The late successional site, L2, for example, is one of the most preserved and structurally complex sites where rare species mainly associated with preserved forests (*C. salvini*, *C. senex)* occur [32], [40]. This site, however, presented a low species richness which, in fact, was reduced to zero during the DS. On the other hand, the highest species richness in the early successional stage of Venezuela can be related to the proximity of a permanent source of water (discussed above). This water source is especially important in attracting species from the subfamily Phyllostominae (the most speciose group in the region), because of its higher concentration of prey. Finally, in the case of Brazil, the positive relationship between assemblage species richness and vegetation structural complexity, is congruent with the findings of Medellín et al. [35], implying that a higher vegetation structural complexity can offer a higher diversity of resources (i.e. food and roosts) allowing for the occurrence of a greater number of species.

Our results indicate that, when characterizing bat responses to succession or other habitat changes, we must extend our focus beyond the analysis of habitat attributes at a local scale (i.e. comparing different types of habitats, quantifying and evaluating the effect of vegetation structural complexity) to a landscape scale. Future studies should include the characterization of landscape attributes (composition and configuration) as they appear to greatly influence the occurrence of vagile species in a particular area [33], [94], [96], [97]. This will allow us to better understand the distribution of phyllostomid bats in anthropogenic landscapes, as well as the ecological processes underlying their assemblages.

## Conclusions

In general, phyllostomid response to habitat change in TDFs showed a high level of regional specificity. This response also showed a marked seasonal specificity as most bat responses were season specific. Climate distinctiveness, the specific ecological requirements and behavior of bat species in each region, the composition and structure of plant assemblages associated with different successional stages, as well as the specific landscape composition and configuration within each region, constituted important sources of variation precluding the detection of clear patterns in this Neotropical ecosystem.

Based on our findings, we conclude that, in tropical seasonal environments, it is imperative to account for seasonal variations in environmental conditions and in the abundance and diversity of resources (emphasizing plant phenological patterns). This will allow us to understand seasonal dynamics in the use of space by phyllostomid bats, especially in those regions suffering from severe forest conversion [33]. The characterization of landscape attributes is also necessary, as the detection of landscape level patterns will allow us to generate models and make predictions that potentially can be extrapolated to different tropical regions. This would provide a better understanding about the ecology and status of bats in the increasingly abundant anthropogenic landscapes.

Finally, findings such as the high inter-annual rate of species turnover in the studied assemblages, indicate that long-term studies are crucial to accurately characterize the phyllostomid assemblages and to identify the potential patterns in their response to habitat modification. The identification of such patterns would constitute an invaluable tool for the preservation of bat biodiversity and the key ecological processes they perform in transformed landscapes. After all “To do science is to search for repeated patterns” [98].

## Supporting Information

Figure S1 **Sampling site scores on PCA axis 1 reflecting vegetation structural complexity in the three study regions.** Study regions: Chamela-Cuixmala Biosphere Reserve (black bars), Unidad de Producción Socialista Agropecuaria Piñero (gray bars) and Mata Seca State Park (white bars). Sampling sites representing different successional stages are: pastures (from P1 to P3), early (from E1 to E3), intermediate (from I1 to I3) and late stage (from L1 to L3).
(TIF)

Figure S2 **Percentage of total species represented by each subfamily (A) and broad guild (B).** Study regions: Chamela Cuixmala Biosphere Reserve in Mexico (Mexico), Unidad de Producción Socialista Agropecuaria Piñero in Venezuela (Venezuela), and Mata Seca State Park in Brazil (Brazil).
(TIFF)

Table S1 **Number of individuals captured in each region during the rainy and the dry season (RS/DS).**
(DOC)

Table S2 **Turnover rate in species composition between consecutive sampling years for each region.**
(DOC)

Table S3 **Seasonal percentage of variation in population, ensemble and assemblage-level parameters associated with the variation of habitat attributes.**
(DOC)

Result S1 **Rank-abundance curves of the sampled phyllostomid assemblages.**
(DOC)

Result S2 **Average values and 95% confidence intervals of the response variables significantly differing among successional stages/seasons.**
(DOC)

Result S3 **Non-metric multidimensional scaling ordinations of sampling sites based on phyllostomid guild composition.**
(DOC)

Result S4 **Non-metric multidimensional scaling ordinations of sampling sites based on phyllostomid species composition.**
(DOC)
