# Supplementary figures and images for: Phyllostomid Bat Occurrence in Successional Stages of Neotropical Dry Forests
Source: PLoS One. 2014 Jan 3;9(1):e84572. doi: 10.1371/journal.pone.0084572 (PMC3880304; doi:10.1371/journal.pone.0084572)

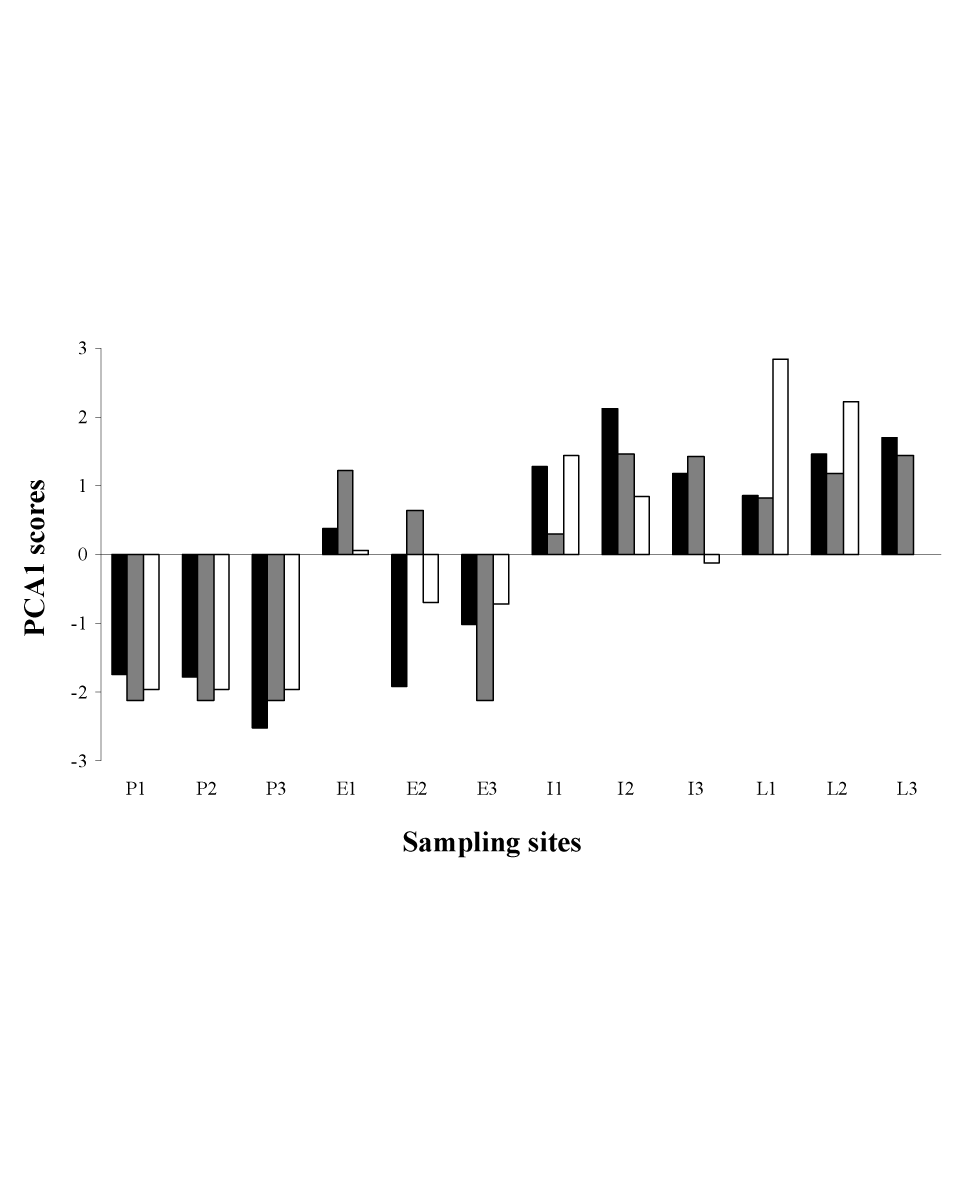

Supplement: Figure S1 — Sampling site scores on PCA axis 1 reflecting vegetation structural complexity in the three study regions. Study regions: Chamela-Cuixmala Biosphere Reserve (black bars), Unidad de Producción Socialista Agropecuaria Piñero (gray bars) and Mata Seca State Park (white bars). Sampling sites representing different successional stages are: pastures (from P1 to P3), early (from E1 to E3), intermediate (from I1 to I3) and late stage (from L1 to L3). (TIF) [file pone.0084572.s001.tif]

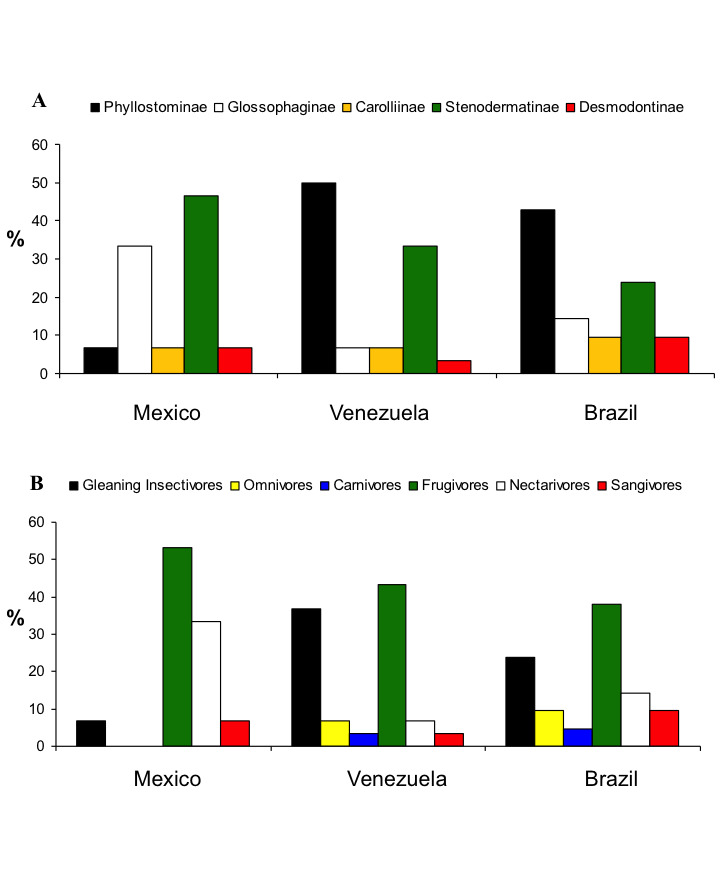

Supplement: Figure S2 — Percentage of total species represented by each subfamily (A) and broad guild (B). Study regions: Chamela Cuixmala Biosphere Reserve in Mexico (Mexico), Unidad de Producción Socialista Agropecuaria Piñero in Venezuela (Venezuela), and Mata Seca State Park in Brazil (Brazil). (TIFF) [file pone.0084572.s002.tiff]
