# Supplementary material for: Phyllostomid Bat Occurrence in Successional Stages of Neotropical Dry Forests
Source: PLoS One. 2014 Jan 3;9(1):e84572. doi: 10.1371/journal.pone.0084572 (PMC3880304; doi:10.1371/journal.pone.0084572)
Supplement: Table S1 — Number of individuals captured in each region during the rainy and the dry season (RS/DS). (DOC) [file pone.0084572.s003.doc]

## Table S1. Number of individuals captured at each region during the rainy and the dry seasons (RS/DS)

| **Region** | **Sampling Sites** | | | | | | | | | | | |
| --- | --- | --- | --- | --- | --- | --- | --- | --- | --- | --- | --- | --- |
| **Mexico** | **Pasture** | | | **Early** | | | **Intermediate** | | | **Late** | | |
| **Species** | **P1** | **P2** | **P3** | **E1** | **E2** | **E3** | **I1** | **I2** | **I3** | **L1** | **L2** | **L3** |
| Desmodontinae |  |  |  |  |  |  |  |  |  |  |  |  |
| *Desmodus rotundus* | 0/- | 0/- | 0/- | 2/0 | 1/0 | 6/1 | 1/5 | 0/2 | 3/10 | 10/5 | 0/0 | 37/37 |
| Glossophaginae |  |  |  |  |  |  |  |  |  |  |  |  |
| *Choeroniscus godmani* | 0/- | 0/- | 0/- | 0/0 | 0/0 | 0/0 | 0/0 | 0/0 | 0/0 | 0/0 | 0/0 | 1/0 |
| *Glossophaga commissarisi* | 0/- | 0/- | 0/- | 3/0 | 0/1 | 15/1 | 0/0 | 0/4 | 0/1 | 0/0 | 0/0 | 2/2 |
| *Glossophaga soricina* | 2/- | 1/- | 0/- | 27/1 | 1/2 | 25/5 | 2/0 | 2/6 | 2/4 | 4/0 | 0/0 | 4/17 |
| *Leptonycteris yerbabuenae* | 0/- | 0/- | 0/- | 12/0 | 0/0 | 15/0 | 0/0 | 1/0 | 1/0 | 1/0 | 0/0 | 0/0 |
| *Musonycteris harrisoni* | 0/- | 0/- | 0/- | 0/0 | 0/0 | 0/0 | 0/0 | 0/0 | 0/0 | 1/0 | 0/0 | 0/0 |
| Phyllostominae |  |  |  |  |  |  |  |  |  |  |  |  |
| *Micronycteris microtis* | 0/- | 0/- | 0/- | 0/0 | 0/0 | 0/0 | 0/0 | 0/0 | 0/0 | 0/0 | 0/0 | 1/0 |
| Carolliinae |  |  |  |  |  |  |  |  |  |  |  |  |
| *Carollia sp* | 0/- | 0/- | 0/- | 0/0 | 0/0 | 0/0 | 0/0 | 0/0 | 0/0 | 0/0 | 0/0 | 7/0 |
| Stenodermatinae |  |  |  |  |  |  |  |  |  |  |  |  |
| *Artibeus jamaicensis* | 0/- | 1/- | 5/- | 5/3 | 8/3 | 7/7 | 11/0 | 17/15 | 4/18 | 5/1 | 1/0 | 62/51 |
| *Artibeus lituratus* | 0/- | 0/- | 1/- | 1/0 | 2/0 | 0/0 | 1/1 | 2/2 | 0/2 | 0/0 | 5/0 | 13/13 |
| *Artibeus phaeotis* | 0/- | 0/- | 0/- | 5/0 | 0/0 | 0/1 | 0/0 | 0/1 | 3/2 | 0/0 | 1/0 | 5/10 |
| *Artibeus watsoni* | 0/- | 1/- | 1/- | 0/0 | 0/0 | 0/3 | 1/0 | 4/3 | 0/0 | 1/0 | 0/0 | 1/0 |
| *Centurio senex* | 0/- | 0/- | 0/- | 0/0 | 0/0 | 0/0 | 0/0 | 0/0 | 0/0 | 0/0 | 1/0 | 0/0 |
| *Chiroderma salvini* | 0/- | 0/- | 0/- | 0/0 | 0/0 | 0/0 | 0/0 | 0/0 | 0/0 | 0/0 | 1/0 | 0/0 |
| *Sturnira lilium* | 0/- | 0/- | 0/- | 0/0 | 1/0 | 1/3 | 0/0 | 1/2 | 0/0 | 0/0 | 0/0 | 3/2 |
| ***Number of individuals*** | 2/- | 3/- | 7/- | 55/5 | 13/6 | 69/21 | 16/6 | 27/37 | 13/37 | 22/8 | 9/0 | 136/132 |
| ***Number of species*** | 1/- | 3/- | 3/- | 7/2 | 5/3 | 6/7 | 5/2 | 6/8 | 5/6 | 6/2 | 5/0 | 11/7 |
| ***Samplings completeness*** | 90/- | 100/- | 95/- | 95/99 | 99/96 | 98/96 | 97/97 | 97/93 | 96/93 | 97/99 | 100/- | 97/98 |

| **Region** | **Sampling Sites** | | | | | | | | | | | |
| --- | --- | --- | --- | --- | --- | --- | --- | --- | --- | --- | --- | --- |
| **Venezuela** | **Pasture** | | | **Early** | | | **Intermediate** | | | **Late** | | |
| **Species** | **P1** | **P2** | **P3** | **E1** | **E2** | **E3** | **I1** | **I2** | **I3** | **L1** | **L2** | **L3** |
| Desmodontinae |  |  |  |  |  |  |  |  |  |  |  |  |
| *Desmodus rotundus* | 0/- | 0/0 | 4/1 | 5/0 | 10/1 | 5/0 | 1/2 | 3/4 | 1/5 | 2/0 | 2/0 | 1/1 |
| Glossophaginae |  |  |  |  |  |  |  |  |  |  |  |  |
| *Glossophaga longirostris* | 1/- | 1/0 | 2/4 | 3/0 | 3/12 | 3/2 | 5/2 | 3/3 | 0/2 | 5/8 | 1/1 | 10/2 |
| *Glossophaga soricina* | 0/- | 0/0 | 0/0 | 0/2 | 2/0 | 0/0 | 0/0 | 2/1 | 0/0 | 1/1 | 0/1 | 1/1 |
| Phyllostominae |  |  |  |  |  |  |  |  |  |  |  |  |
| *Lophostoma brasiliense* | 0/- | 0/0 | 0/0 | 0/0 | 0/0 | 0/0 | 2/2 | 0/4 | 1/1 | 0/1 | 0/1 | 0/2 |
| *Microncyteris hirsuta* | 0/- | 0/0 | 0/0 | 0/0 | 0/0 | 0/0 | 0/0 | 0/0 | 0/1 | 0/1 | 0/2 | 0/0 |
| *Micronycteris megalotis* | 0/- | 1/1 | 0/0 | 0/0 | 0/1 | 1/0 | 1/0 | 3/0 | 0/0 | 1/0 | 1/0 | 0/0 |
| *Micronycteris microtis* | 0/- | 0/0 | 0/0 | 0/0 | 1/0 | 0/0 | 0/0 | 0/0 | 0/0 | 0/0 | 2/0 | 0/0 |
| *Micronycteris minuta* | 1/- | 0/0 | 2/0 | 0/0 | 1/0 | 1/1 | 2/0 | 2/0 | 0/0 | 0/0 | 0/1 | 0/0 |
| *Micronycteris schmidtorum* | 0/- | 0/0 | 0/0 | 1/1 | 0/0 | 1/0 | 0/1 | 3/0 | 0/0 | 0/0 | 0/0 | 1/0 |
| *Mimon bennettii* | 0/- | 0/0 | 0/0 | 0/1 | 0/0 | 0/0 | 0/0 | 0/1 | 0/0 | 0/0 | 0/0 | 0/0 |
| *Mimon crenulatum* | 0/- | 0/0 | 0/0 | 0/0 | 0/0 | 0/0 | 0/0 | 0/0 | 0/0 | 0/3 | 0/0 | 0/3 |
| *Phylloderma stenops* | 0/- | 0/0 | 0/0 | 0/0 | 0/0 | 0/0 | 0/0 | 0/1 | 0/0 | 0/0 | 0/0 | 0/0 |
| *Phyllostomus elongatus* | 9/- | 3/1 | 2/3 | 8/2 | 4/10 | 13/5 | 15/6 | 13/6 | 5/3 | 10/2 | 2/5 | 4/2 |
| *Phyllostomus hastatu*s | 0/- | 0/0 | 0/0 | 0/0 | 1/1 | 13/0 | 2/0 | 0/0 | 0/0 | 0/0 | 0/0 | 2/0 |
| *Tonatia saurophila* | 0/- | 0/0 | 0/0 | 0/0 | 0/1 | 0/0 | 0/0 | 0/0 | 0/0 | 0/0 | 0/1 | 0/0 |
| *Trachops cirrhosus* | 0/- | 0/0 | 1/0 | 1/1 | 2/1 | 2/1 | 1/1 | 0/0 | 1/0 | 0/1 | 0/0 | 0/0 |
| *Trinycteris nicefori* | 0/- | 0/0 | 0/0 | 1/1 | 0/1 | 1/0 | 0/0 | 0/0 | 0/1 | 1/1 | 1/0 | 1/3 |
| *Vampyrum spectrum* | 0/- | 0/0 | 0/0 | 0/0 | 1/0 | 0/0 | 0/0 | 0/0 | 0/0 | 0/0 | 0/0 | 0/0 |
| Carolliinae |  |  |  |  |  |  |  |  |  |  |  |  |
| *Carollia brevicauda* | 1/- | 0/0 | 0/0 | 3/4 | 4/7 | 4/1 | 5/2 | 2/2 | 3/0 | 10/2 | 2/2 | 21/5 |
| *Carollia perspicillata* | 0/- | 0/0 | 0/0 | 1/4 | 1/6 | 2/2 | 6/0 | 4/2 | 0/1 | 4/2 | 0/0 | 7/07 |
| Stenodermatinae |  |  |  |  |  |  |  |  |  |  |  |  |
| *Artibeus jamaicensis* | 0/- | 0/0 | 0/0 | 3/1 | 0/0 | 0/2 | 0/6 | 3/1 | 3/2 | 2/2 | 1/0 | 21/1 |
| *Artibeus lituratus* | 0/- | 1/0 | 1/0 | 1/0 | 0/0 | 0/0 | 0/0 | 0/0 | 0/0 | 0/0 | 0/0 | 0/0 |
| *Chiroderma salvini* | 0/- | 0/0 | 0/0 | 0/0 | 0/0 | 0/0 | 0/0 | 0/0 | 0/0 | 0/1 | 0/0 | 1/0 |
| *Chiroderma villosum* | 0/- | 0/0 | 0/0 | 0/0 | 0/0 | 0/1 | 0/0 | 0/0 | 0/0 | 0/0 | 0/0 | 1/0 |
| *Platyrrhinus helleri* | 0/- | 0/2 | 1/0 | 0/4 | 0/2 | 0/3 | 0/0 | 0/0 | 0/0 | 0/0 | 0/0 | 0/0 |
| *Platyrrhinus. vittatus* | 1/- | 0/0 | 1/0 | 6/4 | 1/5 | 3/0 | 14/21 | 18/4 | 4/0 | 11/8 | 0/1 | 22/1 |
| *Sphaeronycteris toxophyllum* | 1/- | 0/0 | 0/0 | 0/0 | 0/0 | 0/0 | 1/0 | 2/0 | 0/1 | 0/0 | 0/0 | 0/0 |
| *Sturnira lilium* | 2/- | 1/3 | 1/0 | 1/2 | 0/3 | 10/9 | 0/0 | 0/0 | 1/0 | 2/0 | 0/0 | 1/0 |
| *Uroderma bilobatum* | 0/- | 0/0 | 0/3 | 4/11 | 5/17 | 1/3 | 0/2 | 2/0 | 0/0 | 2/1 | 0/0 | 1/0 |
| *Uroderma magnirostrum* | 1/- | 6/0 | 9/0 | 5/2 | 3/6 | 10/6 | 10/2 | 2/0 | 4/0 | 1/0 | 0/0 | 4/0 |
| ***Number of individuals*** | 17/- | 13/7 | 24/11 | 43/40 | 39/74 | 70/36 | 65/47 | 62/30 | 23/17 | 52/35 | 12/12 | 99/29 |
| ***Number of species*** | 8/- | 6/4 | 10/4 | 14/14 | 14/15 | 15/12 | 13/11 | 14/12 | 9/9 | 13/14 | 8/7 | 16/12 |
| ***Samplings completeness*** | 97/- | 96/100 | 97/94 | 98/97 | 100/98 | 95/98 | 99/96 | 97/95 | 96/95 | 96/97 | 98/99 | 99/95 |

| **Region** | **Sampling Sites** | | | | | | | | | | | |
| --- | --- | --- | --- | --- | --- | --- | --- | --- | --- | --- | --- | --- |
| **Brazil** | **Pasture** | | | **Early** | | | **Intermediate** | | | **Late** | | |
| **Species** | **P1** | **P2** | **P3** | **E1** | **E2** | **E3** | **I1** | **I2** | **I3** | **L1** | **L2** | **L3** |
| Desmodontinae |  |  |  |  |  |  |  |  |  |  |  |  |
| *Desmodus rotundus* | 6/15 | 0/1 | 0/2 | 7/13 | 10/9 | 0/9 | 3/5 | 3/1 | 3/3 | 27/20 | 22/22 | 2/13 |
| *Diphylla ecaudata* | 0/0 | 0/0 | 0/2 | 0/2 | 0/1 | 0/2 | 1/1 | 0/0 | 0/1 | 3/2 | 1/2 | 0/3 |
| Glossophaginae |  |  |  |  |  |  |  |  |  |  |  |  |
| *Glossophaga soricina* | 0/0 | 0/0 | 0/1 | 0/2 | 5/6 | 0/1 | 1/3 | 1/5 | 3/1 | 3/2 | 4/1 | 3/9 |
| *Lonchophylla mordax* | 0/3 | 0/1 | 0/0 | 0/1 | 2/1 | 0/0 | 0/1 | 0/0 | 4/0 | 1/1 | 2/0 | 0/0 |
| Phyllostominae |  |  |  |  |  |  |  |  |  |  |  |  |
| *Chrotopterus auritus* | 0/0 | 0/0 | 0/0 | 0/0 | 0/0 | 0/0 | 0/0 | 0/0 | 0/0 | 1/1 | 2/1 | 0/0 |
| *Lophostoma brasiliense* | 0/2 | 0/0 | 0/0 | 0/0 | 0/0 | 0/0 | 0/0 | 1/0 | 0/3 | 0/0 | 0/0 | 0/0 |
| *Micronycteris minuta* | 1/0 | 0/0 | 0/1 | 2/2 | 7/5 | 0/0 | 0/2 | 0/1 | 1/0 | 0/0 | 8/2 | 0/1 |
| *Mimon bennettii* | 0/0 | 0/0 | 0/0 | 0/0 | 1/0 | 0/0 | 0/0 | 0/0 | 0/0 | 1/0 | 1/0 | 0/0 |
| *Mimon crenulatum* | 1/9 | 0/0 | 0/0 | 0/1 | 2/0 | 0/0 | 0/1 | 1/0 | 0/0 | 3/1 | 5/2 | 0/0 |
| *Phylloderma stenops* | 0/0 | 0/0 | 1/1 | 0/1 | 2/1 | 0/2 | 2/1 | 0/2 | 0/0 | 2/2 | 3/2 | 4/0 |
| *Phyllostomus discolor* | 0/13 | 0/0 | 0/0 | 0/0 | 3/1 | 0/0 | 1/0 | 0/0 | 0/1 | 0/27 | 1/1 | 0/1 |
| *Phyllostomus hastatus* | 0/0 | 0/0 | 0/1 | 1/0 | 0/0 | 0/0 | 3/0 | 0/0 | 1/0 | ½ | 1/2 | 0/2 |
| *Tonatia bidens* | 0/0 | 0/0 | 0/0 | 0/1 | 1/1 | 0/0 | 0/0 | 0/1 | 0/0 | 0/0 | 1/0 | 0/0 |
| Carolliinae |  |  |  |  |  |  |  |  |  |  |  |  |
| *Carollia sp* | 2/5 | 2/8 | 1/1 | 14/16 | 7/12 | 1/1 | 2/3 | 4/6 | 3/1 | 9/10 | 10/7 | 10/20 |
| Stenodermatinae |  |  |  |  |  |  |  |  |  |  |  |  |
| *Artibeus lituratus* | 0/0 | 0/0 | 0/0 | 1/0 | 1/2 | 0/1 | 1/0 | 0/0 | 0/1 | 1/1 | 1/2 | 0/0 |
| *Artibeus planirostris* | 2/17 | 1/1 | 0/13 | 2/2 | 9/6 | 1/5 | 1/4 | 0/10 | 3/7 | 7/16 | 14/34 | 4/18 |
| *Chiroderma villosum* | 0/0 | 0/0 | 0/0 | 1/0 | 0/0 | 0/0 | 1/0 | 0/0 | 0/0 | 0/0 | 1/0 | 0/0 |
| *Sturnira lilium* | 0/2 | 0/0 | 0/0 | 0/0 | 0/0 | 0/0 | 0/0 | 0/0 | 0/0 | 0/0 | 0/0 | 0/0 |
| ***Number of individuals*** | 12/66 | 3/11 | 2/22 | 28/41 | 50/46 | 2/21 | 16/21 | 10/26 | 18/19 | 59/85 | 77/79 | 23/70 |
| ***Number of species*** | 5/8 | 2/4 | 2/8 | 7/10 | 12/12 | 2/7 | 10/9 | 5/7 | 7/9 | 12/12 | 16/13 | 5/9 |
| ***Samplings completeness*** | 98/95 | 100/98 | 100/97 | 98/97 | 97/97 | 100/94 | 99/94 | 95/98 | 99/98 | 96/96 | 96/98 | 91/96 |

Sampling sites representing different successional stages are: pastures (from P1 to P3), early (from E1 to E3), intermediate (from I1 to I3) and late stages (from L1 to L3).
