# Supplementary material for: Phyllostomid Bat Occurrence in Successional Stages of Neotropical Dry Forests
Source: PLoS One. 2014 Jan 3;9(1):e84572. doi: 10.1371/journal.pone.0084572 (PMC3880304; doi:10.1371/journal.pone.0084572)
Supplement: Table S2 — Turnover rate in species composition between consecutive sampling years for each region. (DOC) [file pone.0084572.s004.doc]

## Table S2. Turnover rate in species composition between consecutive sampling years for each study region (Mexico, Venezuela and Brazil).

| **Mexico** | **Pair of consecutives sampling years** | |
| --- | --- | --- |
| **Sites** | **2004-2005** | **2005-2006** |
| E1 | 0.27 | **0.50** |
| E2 | − | **0.71** |
| E3 | 0.23 | 0.40 |
| I1 | 0.25 | **0.50** |
| I2 | 0.38 | 0.38 |
| I3 | − | 0.33 |
| L1 | **0.50** | **0.50** |
| L2 | − | **1.00** |
| L3 | 0.29 | 0.13 |
| **Venezuela** | **2007-2008** | **2008-2009** |
| P2 | **0.50** | **0.55** |
| P3 | **0.57** | 0.33 |
| E1 | 0.28 | **0.50** |
| E2 | 0.44 | 0.36 |
| E3 | **0.60** | 0.39 |
| I1 | 0.36 | **0.58** |
| I2 | 0.36 | 0.45 |
| I3 | 0.38 | **0.69** |
| L1 | 0.45 | **0.57** |
| L2 | **0.82** | **0.69** |
| L3 | **0.50** | 0.45 |
| **Brazil** |  |  |
| P1 | 0.33 | 0.27 |
| P2 | 0.33 | **0.50** |
| P3 | 0.33 | **1.00** |
| E1 | 0.41 | 0.45 |
| E2 | 0.47 | 0.25 |
| E3 | 0.33 | 0.27 |
| I1 | **0.50** | **0.64** |
| I2 | 0.45 | 0.45 |
| I3 | 0.45 | **0.64** |
| L1 | 0.20 | 0.33 |
| L2 | 0.26 | **0.53** |
| L3 | 0.33 | 0.38 |

Sampling sites representing different successional stages are: pastures (from P1 to P3), early (from E1 to E3), intermediate (from I1 to I3) and late stages (from L1 to L3). The turnover rate in species composition between consecutive years was quantified by calculating *T* = (*J*+*E*)/(*S_1_*+*S_2_*), where *J* is the number of species exclusively found in the second year, *E* is the number of species exclusively found in the first year, and *S_1_* and *S_2_* are the total number of species during the first and the second year, respectively. The values of this metric vary from 0 (none turnover) to 1 (complete turnover). Turnover rates equal or greater than 0.50 are showed in bold. This metric has been used for similar purposes by Aguirre et al. [1].

## References

1. Aguirre LF, Lens L, van Damme R, Matthysen E (2003) Consistency and variation in the bat assemblages inhabiting two forest islands within a Neotropical savanna in Bolivia. J Trop Ecol 19: 367–374.
