# Supplementary material for: Phyllostomid Bat Occurrence in Successional Stages of Neotropical Dry Forests
Source: PLoS One. 2014 Jan 3;9(1):e84572. doi: 10.1371/journal.pone.0084572 (PMC3880304; doi:10.1371/journal.pone.0084572)
Supplement: Table S3 — Seasonal percentage of variation in population, ensemble and assemblage-level parameters associated with the variation of habitat attributes. (DOC) [file pone.0084572.s005.doc]

## Table S3. Seasonal percentage of variation in population, ensemble and assemblage-level parameters associated to the variation of habitat attributes.

|  |  |  |  | **Explanatory variable** | |
| --- | --- | --- | --- | --- | --- |
| **Response variable** | **Guild** | **n** | ***R^2^_dev_*** | **S_stage_** | **V_struct_** |
| **Mexico** |  |  |  |  |  |
| ***RS*** |  |  |  |  |  |
| ARJAM | F | 9 | 0.109 | 56.014 | 43.986 |
| ARLIT | F | 9 | 0.406 | 84.957 | 15.043 |
| ARPHA | F | 9 | 0.274 | 21.280 | 78.720 |
| GLSOR | N | 9 | 0.795 | **81.649** | 18.351 (-) |
| DEROT | S | 9 | 0.386 | 86.598 | 13.402 |
| F |  | 9 | 0.231 | 57.805 | 42.195 |
| N |  | 9 | 0.723 | **79.907** | 20.093 (-) |
| Species SC_1_ |  | 8 | 0.515 | 80.029 | 19.971 (-) |
| Species SC_2_ |  | 8 | 0.809 | **73.786** | 26.214 (-) |
| Guild SC_1_ |  | 8 | 0.494 | 62.957 | 37.043 |
| Guild SC_2_ |  | 8 | 0.795 | **76.667** | 23.333 |
| Jack1 |  | 9 | 0.450 | 37.919 | 62.081 |
| ***DS*** |  |  |  |  |  |
| ARJAM | F | 9 | 0.148 | 31.247 | 68.753 |
| ARLIT | F | 9 | 0.710 | 40.279 | 59.721 |
| ARPHA | F | 9 | 0.310 | 53.140 | 46.860 |
| GLSOR | N | 9 | 0.035 | 37.213 | 62.787 |
| DEROT | S | 9 | 0.531 | 56.360 | 43.641 |
| F |  | 9 | 0.151 | 32.391 | 67.609 |
| N |  | 9 | 0.033 | 17.259 | 82.741 |
| Species SC_1_ |  | 8 | 0.612 | **83.060** | 16.940 |
| Species SC_2_ |  | 8 | 0.235 | 18.687 | 81.313 |
| Guild SC_1_ |  | 8 | 0.493 | 82.633 | 17.367 |
| Guild SC_2_ |  | 8 | 0.224 | 77.950 | 22.050 |
| Jack1 |  | 9 | 0.141 | 49.571 | 50.428 |

continued…

|  |  |  |  | **Explanatory variable** | |
| --- | --- | --- | --- | --- | --- |
| **Response variable** | **Guild** | **n** | ***R^2^_dev_*** | **S_stage_** | **V_struct_** |
| **Venezuela** |  |  |  |  |  |
| ***RS*** |  |  |  |  |  |
| ARJAM | F | 11 | 0.903 | 8.789 | **91.211** |
| CABRE | F | 11 | 0.662 | 7.631 | **92.369** |
| CAPER | F | 11 | 0.452 | 2.398 | 97.602 |
| PLVIT | F | 11 | 0.483 | 2.264 | **97.736** |
| STLIL | F | 11 | 0.612 | 35.111 | 64.889 (-) |
| URBIL | F | 11 | 0.760 | **73.936** | 26.064 |
| URMAG | F | 11 | 0.440 | 10.602 | 89.398 (-) |
| GLLON | N | 11 | 0.293 | 4.636 | 95.364 |
| PHELO | O | 11 | 0.579 | 82.433 | 17.567 |
| DEROT | S | 11 | 0.690 | **99.086** | 0.914 |
| F |  | 11 | 0.304 | 2.341 | 97.659 |
| N |  | 11 | 0.312 | 2.345 | 97.655 |
| GI |  | 11 | 0.418 | 31.505 | 68.495 |
| O |  | 11 | 0.729 | **98.638** | 1.362 |
| Species SC_1_ |  | 11 | 0.945 | 2.363 | **97.636** |
| Species SC_2_ |  | 11 | 0.411 | 2.718 | 97.281 (-) |
| Guild SC_1_ |  | 11 | 0.192 | 90.685 | 9.315 |
| Guild SC_2_ |  | 11 | 0.858 | **58.686** | **41.314** (-) |
| Jack1 |  | 11 | 0.484 | **90.031** | 9.969 |
| ***DS*** |  |  |  |  |  |
| ARJAM | F | 11 | 0.730 | 88.060 | 11.940 |
| CABRE | F | 11 | 0.707 | 43.213 | 56.787 |
| CAPER | F | 11 | 0.598 | 46.001 | 53.999 |
| PLVIT | F | 11 | 0.388 | 45.905 | 54.095 |
| STLIL | F | 11 | 0.868 | 35.694 | **64.306** (-) |
| URBIL | F | 11 | 0.792 | **98.841** | 1.159 |
| URMAG | F | 11 | 0.847 | **94.272** | 5.728 (-) |
| GLLON | N | 11 | 0.111 | 79.880 | 20.120 |
| PHELO | O | 11 | 0.356 | **96.636** | 3.364 |
| DEROT | S | 11 | 0.772 | 6.683 | 93.317 |
| F |  | 11 | 0.615 | **99.324** | 0.676 |
| N |  | 11 | 0.219 | 48.959 | 51.041 |
| GI |  | 11 | 0.614 | 2.151 | **97.849** |
| O |  | 11 | 0.355 | **96.662** | 3.338 |
| Species SC_1_ |  | 11 | 0.748 | 5.666 | **94.334** |
| Species SC_2_ |  | 11 | 0.187 | 68.993 | 31.007 |
| Guild SC_1_ |  | 11 | 0.336 | 66.824 | 33.176 |
| Guild SC_2_ |  | 11 | 0.438 | 8.227 | **91.773** |
| Jack1 |  | 11 | 0.769 | **69.956** | 30.043 |

continued…

|  |  |  |  | **Explanatory variable** | |
| --- | --- | --- | --- | --- | --- |
| **Response variable** | **Guild** | **n** | ***R^2^_dev_*** | **S_stage_** | **V_struct_** |
| **Brazil** |  |  |  |  |  |
| ***RS*** |  |  |  |  |  |
| ARPLA | F | 11 | 0.667 | 4.548 | 95.452 |
| CAspp | F | 11 | 0.537 | 50.232 | 49.768 |
| PHSTE | F | 11 | 0.236 | 1.390 | 98.610 |
| GLSOR | N | 11 | 0.626 | 39.984 | 60.016 |
| MICRE | GI | 11 | 0.519 | 7.941 | 92.059 |
| MIMIN | GI | 11 | 0.527 | 76.143 | 23.857 |
| DEROT | S | 11 | 0.603 | 1.066 | **98.934** |
| F |  | 11 | 0.649 | 17.290 | 82.710 |
| N |  | 11 | 0.711 | 48.691 | 51.309 |
| S |  | 11 | 0.631 | 1.165 | **98.835** |
| GI |  | 11 | 0.537 | 16.460 | 83.540 |
| O |  | 11 | 0.463 | 52.639 | 47.361 |
| Species SC_1_ |  | 11 | 0.365 | 2.698 | 97.302 (-) |
| Species SC_2_ |  | 11 | 0.743 | 61.826 | 38.173 |
| Guild SC_1_ |  | 11 | 0.437 | 2.461 | 97.539 (-) |
| Guild SC_2_ |  | 11 | 0.529 | 258.500 | 158.500 |
| Jack1 |  | 11 | 0.665 | 1.763 | **98.237** |
| ***DS*** |  |  |  |  |  |
| ARPLA | F | 11 | 0.648 | 46.314 | 53.686 |
| CAspp | F | 11 | 0.537 | 66.407 | 33.592 |
| PHSTE | F | 11 | 0.393 | 47.561 | 52.439 |
| GLSOR | N | 11 | 0.554 | **84.394** | 15.606 |
| MICRE | GI | 11 | 0.341 | 88.855 | 11.145 (-) |
| MIMIN | GI | 11 | 0.279 | 95.206 | 4.794 |
| DEROT | S | 11 | 0.653 | 2.062 | 97.938 |
| F |  | 11 | 0.700 | 10.950 | 89.050 |
| N |  | 11 | 0.375 | **97.417** | 2.583 |
| S |  | 11 | 0.673 | 5.333 | 94.667 |
| GI |  | 11 | 0.039 | 8.557 | 91.443 (-) |
| O |  | 11 | 0.706 | 45.337 | 54.663 |
| Species SC_1_ |  | 11 | 0.274 | 2.336 | 97.663 |
| Species SC_2_ |  | 11 | 0.269 | 56.951 (-) | 156.951 |
| Guild SC_1_ |  | 11 | 0.264 | 18.636 | 81.364 |
| Guild SC_2_ |  | 11 | 0.447 | 0.119 (-) | 100.119 |
| Jack1 |  | 11 | 0.679 | 6.876 | **93.124** |

Seasons: rainy (RS) and dry season (DS). Parameters at population-level: capture rate (individuals/night) as indicator of local abundance of the species *Artibeus jamaicensis* (ARJAM), *A. planirostris* (ARPLA), *A. lituratus* (ARLIT), *A. phaeotis* (ARPHA), *Platyrrhinus vittatus* (PLVIT), *Sturnira lilium* (STLIL)*, Uroderma bilobatum* (URBIL), *Uroderma magnirostrum* (URMAG), *Carollia brevicauda* (CABRE), *C. perspicillata* (CAPER), *Carollia* spp (CAspp), *Glossophaga soricina* (GLSOR), *G. longirostris* (GLLON), *Mimon crenulatum* (MICRE), *Micronycteris minuta* (MIMIN), *Phyllostomus elongatus* (PHELO), *Phylloderma stenops* (PHSTE), and *Desmodus rotundus* (DEROT). Parameters at ensemble-level: capture rate (individuals/night) as indicator of local abundance of frugivorous (F), nectarivorous (N), sanguivorous (S), gleaning insectivorous (GI), and omnivorous bats (O). Parameters at assemblage-level: scores of the first (Species SC_1_) and second (Species SC_2_) ordination axis reflecting assemblages’ dissimilarities in species composition; scores of the first (Guild SC_1_) and second (Guild SC_2_) ordination axis reflecting assemblages’ dissimilarities in guild composition; and the species richness estimated by using the first-order jackknife estimator (Jack1). Explanatory variables: successional stage (Sstage) and scores of the first ordination axis reflecting sampling sites’ dissimilarities in vegetation structural complexity (Vstruct). n: number of sampling sites. *R^2^_dev_* is the fraction of the total deviance explained by a model considering all explanatory variables when the Poisson error distribution was used and *R^2^* when the normal error distribution was used. Significant relationships according to the randomization test appear in bold. Negative relationships are shown in parentheses.
