# Supplementary material for: Phyllostomid Bat Occurrence in Successional Stages of Neotropical Dry Forests
Source: PLoS One. 2014 Jan 3;9(1):e84572. doi: 10.1371/journal.pone.0084572 (PMC3880304; doi:10.1371/journal.pone.0084572)
Supplement: Result S1 — Rank-abundance curves of the sampled phyllostomid assemblages. (DOC) [file pone.0084572.s006.doc]

## Result S1. Rank-abundance curves of the sampled phyllostomid assemblages.

##
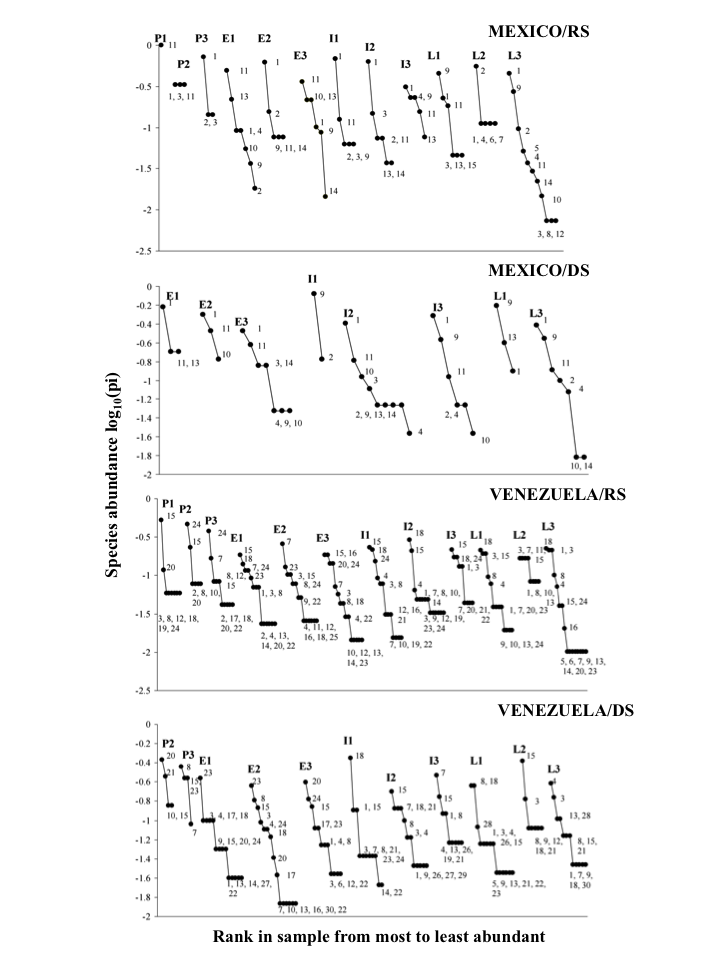


##
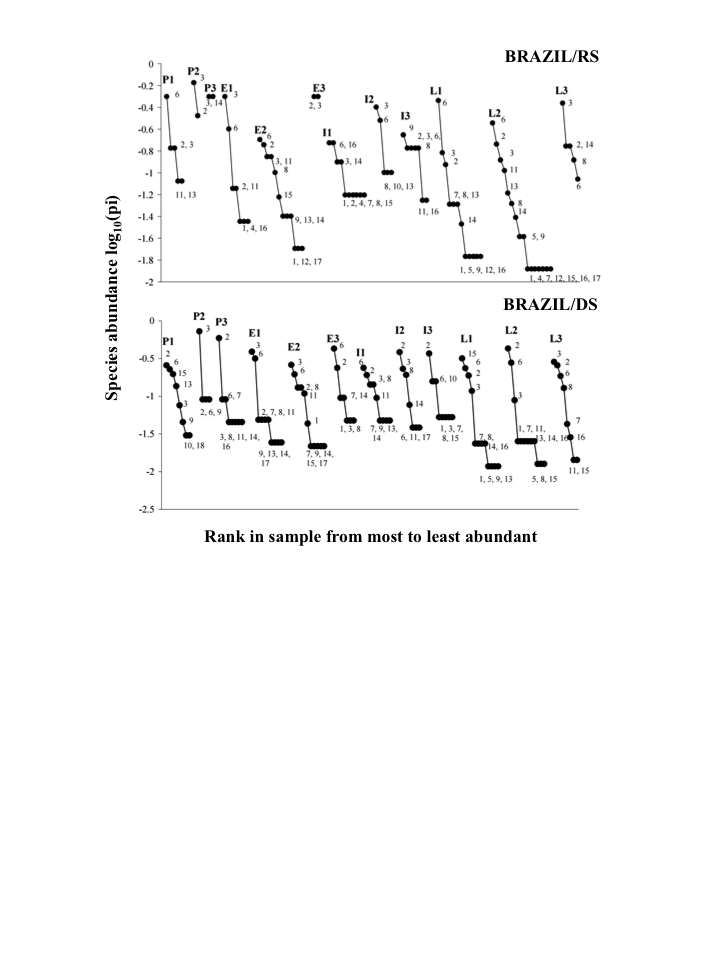


Sampling sites representing different successional stages are: pastures (from P1 to P3), early (from E1 to E3), intermediate (from I1 to I3) and late stage (from L1 to L3). Seasons: rainy season (RS), and dry season (DS). Numbers represent the captured species according to the following lists in which the species subfamily (St: Stenodermatinae, Gl: Glossophaginae, Ph: Phyllostominae, Cr: Carolliinae, Ds: Desmodontinae) and foraging guild (F: Frugivore, N: Nectarivore, GI: Gleaning Insectivore, C: Carnivore, O: Omnivore, S: Sanguivore) are also specified in parentheses.

Species list for Mexico: 1) *Artibeus jamaicensis* (St, F), 2) *A. lituratus* (St, F), 3) *A. watsoni* (St, F), 4) *A. phaeotis* (St, F), 5) *Carollia sp* (Cr, F), 6) *Centurio senex* (St, F), 7) *Chiroderma salvini* (St, F), 8 ) *Choeroniscus godmani* (Gl, N), 9) *Desmodus rotundus* (Ds, S), 10) *Glossophaga commissarisi* (Gl, N), 11) *G. soricina* (Gl, N), 12) *Micronycteris microtis* (Ph, GI), 13) *Leptonycteris yerbabuenae* (Gl, N), 14) *Sturnira lilium* (St, F) and 15) *Musonycteris harrisoni* (Gl, N).

Species list for Venezuela: 1) *Artibeus jamaicensis* (St, F), 2) *A. lituratus* (St, F), 3) *Carollia brevicauda* (Cr, F), 4) *C. perspicillata* (Cr, F), 5) *Chiroderma salvini* (St, F), 6) *C. villosum* (St, F), 7) *Desmodus rotundus* (Ds, S), 8) *Glossophaga longirostris* (Gl, N), 9) *G. soricina* (Gl, N), 10) *Micronycteris megalotis* (Ph, GI), 11) *M. microtis* (Ph, GI), 12) *M. minuta* (Ph, GI), 13) *Trinycteris nicefori* (Ph, GI), 14) *M. schmidtorum* (Ph, GI), 15) *Phyllostomus elongates* (Ph, O), 16) *P. hastatu*s (Ph, O), 17) *Platyrrhinus helleri* (St, F)*,* 18) *P. vittatus* (St, F), 19) *Sphaeronycteris toxophyllum* (St, F), 20) *Sturnira lilium* (St, F), 21) *Lophostoma brasiliense* (Ph, GI), 22) *Trachops cirrhosus* (Ph, GI), 23) *Uroderma bilobatum* (St, F), 24) *U. magnirostrum* (St, F), 25) *Vampyrum spectrum* (Ph, C), 26) *Microncyteris hirsuta* (Ph, GI), 27) *Mimon bennettii* (Ph, GI), 28) *M. crenulatum* (Ph, GI), 29) *Phylloderma stenops* (Ph, F), and 30) *Tonatia saurophila* (Ph, GI).

Species list for Brazil: 1) *Artibeus lituratus* (St, F), 2) *A. planirostris* (St, F), 3) *Carollia spp*, grouping *C. brevicauda* and *C. perspicillata* capture data (Cr, F), 4) *Chiroderma villosum* (St, F), 5) *Chrotopterus auritus* (Ph, C), 6) *Desmodus rotundus* (Ds, S), 7) *Diphylla ecaudata* (Ds, S), 8) *Glossophaga soricina* (Gl, N), 9) *Lonchophylla mordax* (Gl, N), 10) *Lophostoma brasiliense* (Ph, GI), 11) *Micronycteris minuta* (Ph, GI), 12) *Mimon bennettii* (Ph, GI), 13) *M. crenulatum* (Ph, GI), 14) *Phylloderma stenops* (Ph, F), 15) *Phyllostomus discolor* (Ph, O), 16) *P. hastatus* (Ph, O), 17) *Tonatia bidens* (Ph, GI), and 18) *Sturnira lilium* (St, F).
