# Supplementary material for: Phyllostomid Bat Occurrence in Successional Stages of Neotropical Dry Forests
Source: PLoS One. 2014 Jan 3;9(1):e84572. doi: 10.1371/journal.pone.0084572 (PMC3880304; doi:10.1371/journal.pone.0084572)
Supplement: Result S2 — Average values and 95% confidence intervals of the response variables significantly differing among successional stages/seasons. (DOC) [file pone.0084572.s007.doc]

## Result S2. Average values and 95% confidence intervals of the response variables significantly differing among successional stages/seasons.

**MEXICO**

**MEXICO**

**VENEZUELA**

**VENEZUELA**

**HP**

**VENEZUELA**

**VENEZUELA**

**VENEZUELA**

**BRAZIL**

Successional stages: pasture (P), early (E), intermediate (I) and late stage (L).

*Response variables of Mexico*

Response variable at the population-level: capture rate (individuals/night) as indicator of local abundance of the species *Glossophaga soricina* (GLSOR) during the rainy season (RS). Response variable at the ensemble-level: capture rate of nectarivores (N) during the RS. Response variables at the assemblage-level: scores of the ordination axes reflecting assemblages’ dissimilarities in species composition (Species NMDS2, Species NMDS1) and guild composition (Guild NMDS2) during the RS and the dry season (DS).

*Response variables of Venezuela*

Response variables at the population-level: capture rate of *Uroderma bilobatum* (URBIL), *Uroderma magnirostrum* (URMAG), *Desmodus rotundus* (DEROT), and *Phyllostomus elongatus* (PHELO) during the RS and DS. Response variables at the ensemble-level: capture rate of the omnivores (O) and frugivores (F) during the RS and the DS. Response variables at the assemblage-level: species richness during the RS and DS estimated by using the first-order jackknife estimator (Jack1) and scores of the second ordination axis reflecting assemblages’ dissimilarities in guild composition (Guild NMDS2) during the RS.

*Response variables of Brazil*

Response variable at the population-level: capture rate of *G. soricina* during the DS. Response variable at the ensemble-level: capture rate of nectarivores during the DS.
