# Supplementary material for: Phyllostomid Bat Occurrence in Successional Stages of Neotropical Dry Forests
Source: PLoS One. 2014 Jan 3;9(1):e84572. doi: 10.1371/journal.pone.0084572 (PMC3880304; doi:10.1371/journal.pone.0084572)
Supplement: Result S3 — Non-metric multidimensional scaling ordinations of sampling sites based on phyllostomid guild composition. (DOC) [file pone.0084572.s008.doc]

## Result S3. Non-metric multidimensional scaling ordinations of sampling sites, based on guild composition.

**Stress**: 0.005

**MEXICO – RS**

**NMDS2**

**Stress**: 0.006

**MEXICO – DS**

**Stress**: 3.469

**NMDS2**

**NMDS1**

**Stress**: 6.968

**VENEZUELA – RS**

**VENEZUELA – DS**

**Stress**: 5.208

**NMDS2**

**NMDS1**

**Stress**: 8.313

**BRAZIL – RS**

**BRAZIL – RS**

Seasons: rainy season (RS), and dry season (DS). Sampling sites representing different successional stages are: pastures (from P1 to P3), early (from E1 to E3), intermediate (from I1 to I3) and late stage (from L1 to L3). Ordination axes: first (NMDS1) and second axis (NMDS2). The stress-value corresponding each ordination appears on top of the graphs. Guilds: Gleaning insectivores (GI), frugivores (F), nectarivores (N), omnivores (O), carnivores (C), and sanguivores (S).
