# Supplementary material for: Phyllostomid Bat Occurrence in Successional Stages of Neotropical Dry Forests
Source: PLoS One. 2014 Jan 3;9(1):e84572. doi: 10.1371/journal.pone.0084572 (PMC3880304; doi:10.1371/journal.pone.0084572)
Supplement: Result S4 — Non-metric multidimensional scaling ordinations of sampling sites based on phyllostomid species composition. (DOC) [file pone.0084572.s009.doc]

**Result S4.** Non-metric multidimensional scaling ordinations of sampling sites, based on phyllostomid species composition.

**MEXICO – RS**

**Stress**: 2.081

**NMDS2**

**MEXICO – DS**

**NMDS1**

**Stress**: 0.005

**Stress**: 10.858

**VENEZUELA – RS**

**NMDS2**

**NMDS1**

**Stress**: 13.187

**VENEZUELA – DS**

**Stress**: 9.167

**BRAZIL– RS**

**NMDS2**

**NMDS1**

**Stress**: 8.995

**BRAZIL – DS**

Seasons: rainy season (RS), and dry season (DS). Sampling sites representing different successional stages are: pastures (from P1 to P3), early (from E1 to E3), intermediate (from I1 to I3) and late stage (from L1 to L3). Ordination axes: first (NMDS1) and second axis (NMDS2). The stress-value corresponding each ordination appears on top of the graphs. Numbers represent captured species according to the following lists.

Species list for Mexico: 1) *Artibeus jamaicensis*, 2) *A. lituratus*, 3) *A. watsoni*, 4) *A. phaeotis*, 5) *Carollia sp*, 6) *Centurio senex*, 7) *Chiroderma salvini*, 8 ) *Choeroniscus godmani*, 9) *Desmodus rotundus*, 10) *Glossophaga commissarisi*, 11) *G. soricina*, 12) *Micronycteris microtis*, 13) *Leptonycteris yerbabuenae*, 14) *Sturnira lilium*) and 15) *Musonycteris harrisoni*.

Species list for Venezuela: 1) *Artibeus jamaicensis*, 2) *A. lituratus*, 3) *Carollia brevicauda*, 4) *C. perspicillata*, 5) *Chiroderma salvini*, 6) *C. villosum*, 7) *Desmodus rotundus*, 8) *Glossophaga longirostris*, 9) *G. soricina*, 10) *Micronycteris megalotis*, 11) *M. microtis*, 12) *M. minuta*, 13) *Trinycteris nicefori*, 14) *M. schmidtorum*, 15) *Phyllostomus elongates*, 16) *P. hastatu*s, 17) *Platyrrhinus helleri,* 18) *P. vittatus*, 19) *Sphaeronycteris toxophyllum*, 20) *Sturnira lilium*, 21) *Lophostoma brasiliense*, 22) *Trachops cirrhosus*, 23) *Uroderma bilobatum*, 24) *U. magnirostrum*, 25) *Vampyrum spectrum*, 26) *Microncyteris hirsuta*, 27) *Mimon bennettii*, 28) *M. crenulatum*, 29) *Phylloderma stenops*, and 30) *Tonatia saurophila*.

Species list for Brazil: 1) *Artibeus lituratus*, 2) *A. planirostris*, 3) *Carollia spp*, grouping *C. brevicauda* and *C. perspicillata* capture data, 4) *Chiroderma villosum*, 5) *Chrotopterus auritus*, 6) *Desmodus rotundus*, 7) *Diphylla ecaudata*, 8) *Glossophaga soricina*, 9) *Lonchophylla mordax*, 10) *Lophostoma brasiliense*, 11) *Micronycteris minuta*, 12) *Mimon bennettii*, 13) *M. crenulatum*, 14) *Phylloderma stenops*, 15) *Phyllostomus discolor*, 16) *P. hastatus*, 17) *Tonatia bidens*, and 18) *Sturnira lilium*.
